# In Silico Studies on Sennidines—Natural Dianthrones from Senna

**DOI:** 10.3390/biology10060468

**Published:** 2021-05-26

**Authors:** Sebastian Szymanski, Irena Majerz

**Affiliations:** Faculty of Pharmacy, Wroclaw Medical University, Borowska 211a, 50-556 Wroclaw, Poland; sebastian.szymanski@umed.wroc.pl

**Keywords:** sennidines, conformation, hydrogen bond, QTAIM, NCI

## Abstract

**Simple Summary:**

The study determines the spatial structure and intramolecular interactions of sennidines—natural pharmaceutical substances present in *Senna* species. The calculations predict many sennidin conformers with similar energy but the gauche conformation will be present in the plant material. The lowest energy structure that is most likely to be found in plant material is characterized by the presence of OHO hydrogen bonds formed by hydroxyl groups and carbonyl oxygen. The sanidin molecule can be easily breakdown into monoanthrones because of elongation of the single C-C bond linking the anthrone moieties and reduced bond dissociation energy. The work contains data on theoretical, vibrational and electron excitation spectra, which can be used in the analysis of experimental samples.

**Abstract:**

The rapid development of technology allows for more accurate research of biological systems with the use of in silico methods. One of the tools is the quantum-chemical method used for determining the structure, properties and interactions of molecules of great pharmacological importance. The accuracy of theoretical models is increasing and can be a real help in biology, chemistry and pharmacy. The aim of the study is to determine the spatial structure and intramolecular interactions of sennidines—natural pharmaceutical substances present in *Senna* species. Calculations carried out in the gas-phase and in the solvent model, compared with the available experimental data indicate the possibility of sennidines to form conformers. QTAIM and NCI analysis suggests the presence of many intramolecular interactions in the sennidin structure. Taking into account the lowest energy optimized structure, it can be predicted that the sennidin in the gauche conformation will be present in the plant material. The single C-C bond connecting the anthrone moieties is elongated and its reduced Bond Dissociation Energy (BDE) could be the cause of an easy breakdown of the sennidin molecule into monoanthrones. This work contains data on theoretical, vibrational and electron excitation spectra, which can be used in the analysis of experimental samples.

## 1. Introduction

Sennidines belong to a group of compounds composed of double-anthrone moiety.

**Scheme 1:**
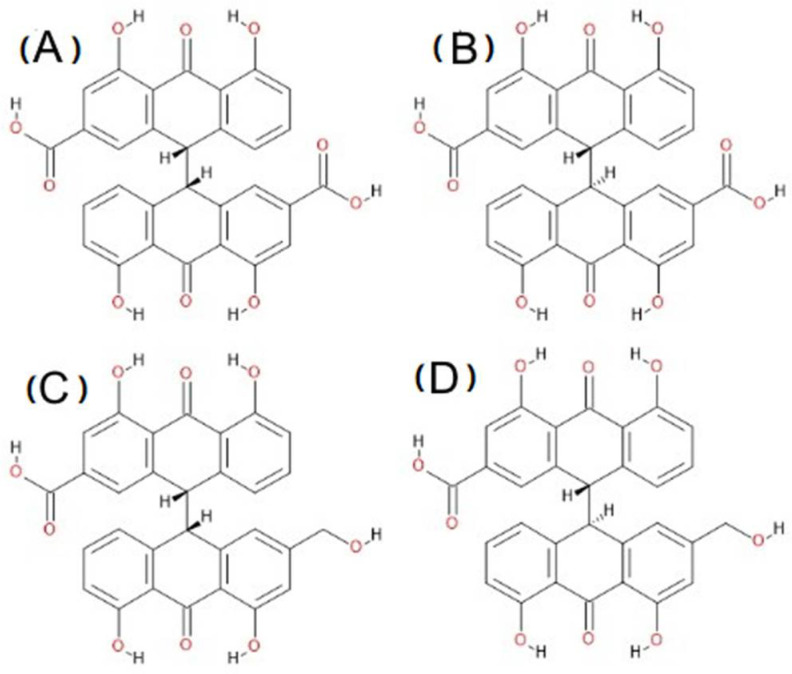
Molecular structures of sennidin (**A**–**D**).

There are four sennidin structures shown in Scheme 1. The common part of all of the sennidines is a double-anthrone moiety connected by a single C-C bond. Two specific regions can be distinguished in the sennidin molecule. The “peri” region, which includes two hydroxyl groups directed to carbonyl oxygen, and the “bay” region, which is located in the empty space between two anthron moieties. There are COOH or CH_2_OH groups in the “bay” region, depending on the sennidin type. The structure of sennidin is characterized by a number of substituents. Substitution allows for the formation of strong OHO hydrogen bonding [1]. The sennidin structure is characterized by the rotation of the anthron moieties. It is possible to distinguish RR and RS isomers of sennidines. In addition, substitution at the “bay” region of the molecule with a COOH or CH_2_OH group produces four sennidin structures, ultimately named sennidin A–D. The ability of sennidines to form conformers and easy breakdown of the single C-C bond between anthron moieties [2] has not been examined sufficiently.

Monoantrons obtained after breaking down the single C-C bond also affect some of the other properties of sennidin, such as antidiabetic [3]. Sennidines occur naturally in Senna plants [4]. The number of *Senna* species is estimated at 260–350, of which nearly 50 are grown to obtain plant material [5]. One of them is *Senna alexandrina* [6]. Senna plants are used as a natural laxative and to fight constipation [7]. The laxative effect of Senna plants is associated with the presence of sennidines [8] and is mainly caused by their decomposition products formed in the intestines [9,10,11].

The widespread availability of Senna laxatives helps patients suffering from chronic constipation who are using opioid therapy and as a laxative [7]. However, it carries the risk of abuse. Some studies have indicated the carcinogenic nature of anthron compounds in the gastrointestinal tract during chronic use [12,13], while other studies have failed to show the effect [14,15]. Sennidin is also an interesting antitumor agent—sennidin A, labeled with iodine 131, can be used to directly target tumor cells. Due to the high affinity and accumulation of sennidin in dead tumor tissue, it is possible to image and kill residual tumor cells. The advantage of using sennidin is its specific accumulation and retention in cancerous tissue and rapid removal from healthy cells [16]. Due to the high affinity of sennidin for dead cells, it can be used to treat of myocardia. Sennidin, when accumulated in degenerated myocardial tissue, allows its selective destruction [17,18]. In plant material, sennidines are present in glycosidic forms, called sennosides. The total sennidin content in the *Senna* species ranges from 0.08% to 2.62%, calculated as sennoside D—one of the four glycosidic form of sennidin [19,20]. The content of sennosides may vary depending on plant nutrition and time when the leaves are picked [21,22]. Extraction of pure sennidines from plant material is a multi-stage and time-consuming process, so a fast and efficient synthesis of sennidin aglycone has been proposed [23]. The bioavailability of dianthrons is low, and only the products resulting from the breakdown of the sennidin molecule into monoanthrones have a laxative effect [24]. The metabolism of sennosides and their mechanism of action in the gastrointestinal tract have been described and are well known [9,25].

Although the crystal structures of dianthrones are known [26,27,28], the structures of sennidines have not been determined so far and in the case of dianthrones there are many structural problems to be solved. The basic question is the mutual arrangement of the two parts of the dianthrone. The results of structural [26,27,28] and theoretical [29] research show that a gauche form is a typical arrangement; however, for dianthrone analogues anti stacking [27] is also possible. Other structural problems are related to unsymmetrically substituted dianthrones and their cis/trans conformation. In the case of sennidines, except the question of the interaction between the anthrone parts, arises the question about the possibility of formation of intramolecular hydrogen bonds between the groups substituted in both parts of the dianthrone.

The purpose of this paper is to apply computational methods to determine the molecular structure of sennidin and to perform conformational analysis of the sennidin molecule. Studies into the structure of sennidin provide information about its possible intramolecular interactions. Data obtained from them may be helpful in further research on the pharmacological properties of sennidines, which result directly from their geometrical and electronic structure. Conformational analysis based on rotation around the C-C single carbon bond has been performed for the structures presented in Scheme 1. It would be interesting to examine whether there are possible intramolecular interactions between substituents for anthrone moieties and how such interactions affect the structure and energy of individual sennidines.

## 2. Materials and Methods

General Experimental Procedures. Conformational analysis and the optimization of the sennidin structures was performed by means of the Gaussian16 software [30]. The optimization was performed at the DFT B3LYP/6-311++G**-DG3 level with Grimme dispersion [31]. The solvent PCM model was calculated using the SCRF command implemented in the Gaussian package with the cavity radius and electric permittivity default for water and ethanol. All of the optimized structures correspond to the local minima on the potential-energy surface. The QTAIM analysis was carried out with the AIMALL program [32]. Non-covalent interactions were investigated with the NCI [33] method. The ACID program was used to describe the delocalization of the electrons [34]. UV–VIS spectra and orbital analysis was performed as a single point calculation using the ADF2019 program [35] with the same method as used during Gaussian16 optimization. Singlet–singlet excitations for ten excited states were computed using Davidson method. The UV–VIS absorption spectra were fitted using Gaussian functions with a width of 50 nm.

## 3. Results and Discussion

Four sennidin structures, named sennidin A–D (Scheme 1) are known. They occur naturally in plant material, but their structure and geometry are not precisely determined. The “peri” region of the anthrone moieties remains similar in all of the sennidines. Substitution in the “bay” region with COOH or CH_2_OH groups differentiates molecules into sennidin A—a structure with two carboxyl groups, and sennidin C—a structure with one carboxyl and one CH_2_OH group. Another difference, which doubles the number of sennidin structures, is the RR and RS conformation of the sennidin molecules within the C-C single bond that connects the anthrone moieties. Sennidin A is the RR configuration of the double-anthrone moiety with two carboxyl groups, while sennidin B represents the RS configuration of the structure. Sennidin C is the RR configuration of the double-anthrone moiety with one COOH and one CH_2_OH group and the RS conformation of the structure represents sennidin D. Despite multiple sennidin A–D conformers, the problem of configuration in sennidin molecules is an important issue. Because of the presence of the C10(sp^3^), C10′(sp^3^) carbon atoms, forming the single C-C bond and the presence of the methoxy and carboxylic group, the A, B and C, D pairs of sennidin are called diastereoismers. Sennidin A and C cannot convert to sennidin B and D without the interference with chemical bonds. The optimization performed on the initial structures maintains the configurations for all sennidin structures. The configurations are 10R,10′R for sennidin A and C and 10R,10′S for sennidin B and D.

The rotation of the anthrone moieties can lead to intramolecular interactions of the substituents in “peri” and “bay” regions of sennidin. The nature of the intramolecular interactions is unexplored and may affect the structure of the double-anthrone moiety.

### 3.1. Conformational Analysis of Sennidines

**Figure 1:**
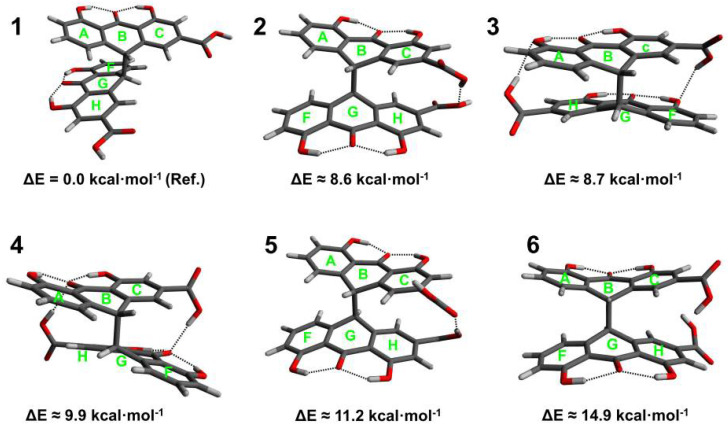
Conformers (**1**–**6**) of sennidin A.

**Figure 2:**
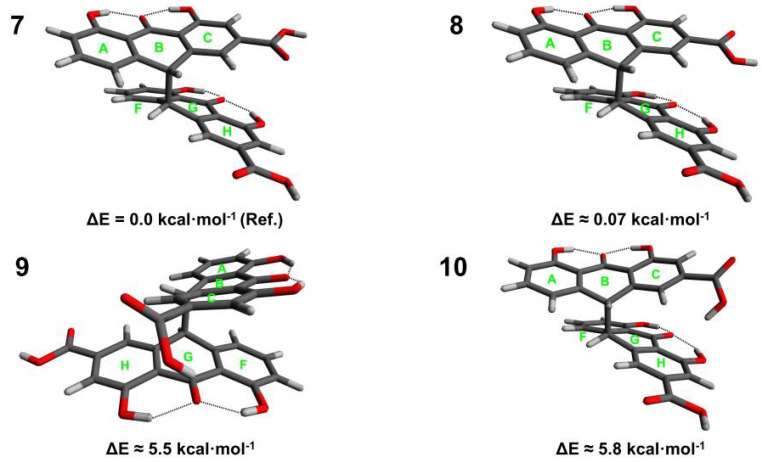
Conformers (**7**–**10**) of sennidin B.

**Figure 3:**
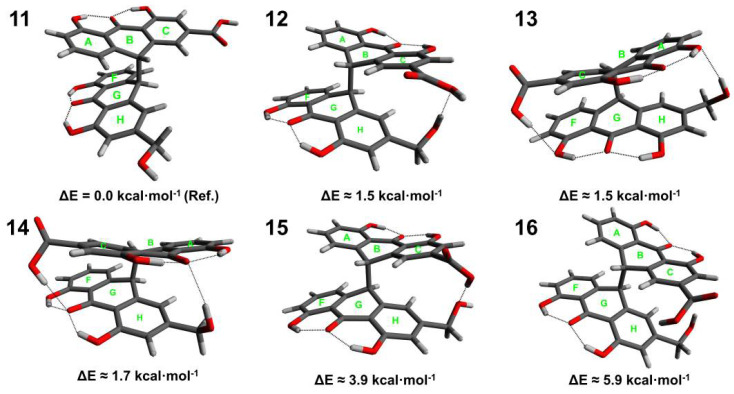
Conformers (**11**–**16**) of sennidin C.

**Figure 4:**
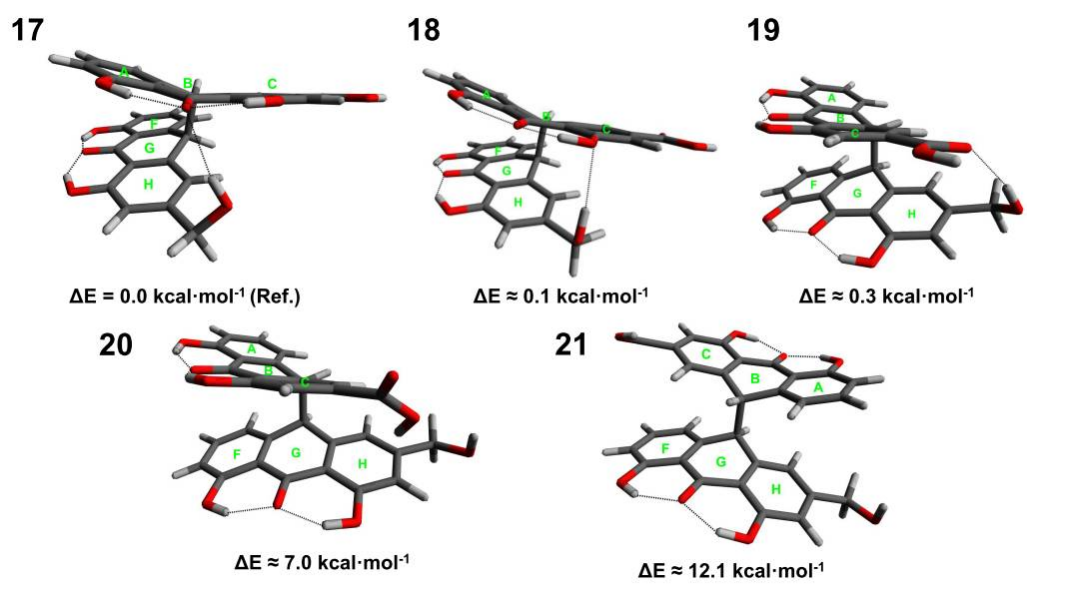
Conformers (**17**–**21**) of sennidin D.

Optimization of the sennidin molecule has been performed for the structures presented in Scheme 1. For sennidin A–D, twenty-one structures showing energy differences have been chosen and for all of them, structure optimization has been performed. Optimized structures corresponding to the local minima of energy with zero-point correction are presented in Figure 1, Figure 2, Figure 3 and Figure 4.

Six low energy conformers have been obtained for sennidin A. The maximum difference in energy is 14.9 kcal∙mol^−1^. Structure 1, with the lowest energy, corresponds to the formation of the anthrone moieties characterized by no intramolecular interactions. The structure is characterized by separation of the substituents in the “bay” region (gauche conformation). In turn, conformer 6, with the highest energy, corresponds to the close position of the substituents in the “bay” region of the molecule (anti conformation). For such conformation, no hydrogen bond between carboxyl groups has been observed. An interesting conformation of structure 4 shows possible triple OHO intramolecular interaction between the substituents.

Four conformers for the sennidin B are characterized by an energy difference of 5.8 kcal∙mol^−1^, and rotation of the carboxyl group is responsible for the energy changes. The lowest energy conformer 7 is characterized by a non-stacking arrangement of the anthrone moieties. There are no interactions between the carboxyl group and the substituents in the “peri” region.

The structure of sennidin C can be described with 6 conformers (11–16). The maximum energy difference is 5.9 kcal∙mol^−1^. Conformer 11, with the lowest energy, is characterized by a non-stacking arrangement of the anthrone moieties. The substituents in “bay” region are arranged parallel to the plane of the C and H rings. A number of intramolecular interactions in the structure of sennidin C can be observed. Conformers 11–14 show similar energy in the range of 1.5–1.7 kcal∙mol^−1^. Despite the stacking of the anthrone moieties, it can be assumed that formation of intramolecular interactions would reduce the energy of the system. However, the distance between the anthrone moieties can introduce repulsive and dispersive interactions, so the overall energetic effect of the molecule is ambiguous.

Five conformers with significant energy differences have been obtained for sennidin D. For conformers 17–19, the energy difference is less than 0.5 kcal∙mol^−1^, which suggests that the structures might exist in plant material. Structure 17 in gauche conformation is additionally stabilized by the formation of an intramolecular O-H⋯O hydrogen bond. The conformer with the lowest energy is characterized by a triple OHO hydrogen bonding to the carbonyl oxygen in the “peri” region of the molecule. Interestingly, structure 21, characterized by a non-stacking arrangement, shows the highest energy. It is characteristic that for the analyzed sennidin A–C structures in the lowest energy, the anthrone moieties are not linked by a hydrogen bond. The number of the obtained structures for individual conformers varies because the possible intramolecular interactions and geometry constraints influence the potential-energy surface and energy minima for every sennidin. For the investigated sennidines, many local energy minima structures can be obtained, but usually the energy difference with respect to the reference structure is relatively high if the intramolecular hydrogen bonds between the anthrone parts are formed.

**Figure 5:**
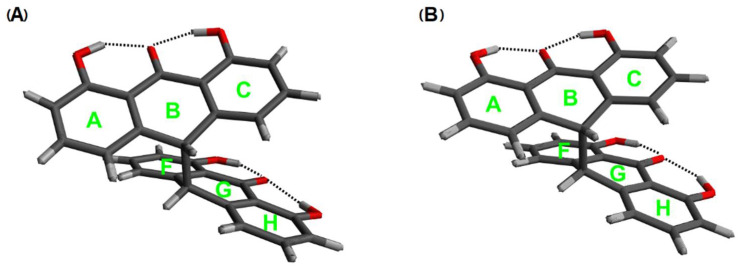
Comparison of the crystal (**A**) and optimized (**B**) structure of the double-anthrone moiety.

Figure 5 shows the crystal structure of the double-anthrone moiety [26] and the structure optimized by the same method as the sennidin molecules. Due to insufficient literature data on the structure of sennidines, the optimized conformers were compared with the double-anthrone moiety that has no substituents in the “bay” region—1,1′,8,8′-tetrahydroxybianthrone. The structural parameters of the sennidin conformers are collated in Table 1 and Table 2. The A and B structures are presented in Figure 5.

### 3.2. Analysis of Geometry of Sennidin Structures

The main geometrical parameter for the sennidin anthrone moieties is the angle between the planes of the A–C and F–H rings (Figure 1, Figure 2, Figure 3 and Figure 4) because it is a measure of the deformation of the anthrone moieties in the sennidin molecule. It may be expected that this deformation results directly from the intramolecular interactions in sennidin structure. The interplanar angles for the sennidin A–D are summarized in Table 1. Structure 1, without any intramolecular hydrogen bonds between the anthrone parts, corresponds to the interplanar A–C angle of 17.7 degrees. Structure 3, showing the interactions between the carboxyl groups and hydroxyl groups in the “peri” region of the molecule, corresponds to the angle of 11.9 degrees. The dual system of the intramolecular OHO hydrogen bonding linking both anthrone parts causes deformation of the anthrone moieties of about 5.8 degrees. In turn, the intramolecular hydrogen bond between the carboxyl groups and the carbonyl oxygen (structure 4) does not affect the inter-planar angle as much as in the previous case.

Changes in the geometric parameters are connected with the formation of hydrogen bonds in the sennidin molecule in which two types of the OHO hydrogen bond exist. The OHO hydrogen bonds in the “peri” region of the molecules show a similar length and angle for most of the sennidines (Table 2). There is no energetically privileged structure characterized by the breaking of the hydrogen bonds in the “peri” region of sennidines. Another type of interaction in the sennidin molecules is the intramolecular OHO hydrogen bond between two anthrone moieties. The bond is created between the COOH or CH_2_OH groups and the substituents in the “peri” region of the molecule. The bond is characterized by a larger distance and a bigger OHO angle. The angles and bond lengths for all structures are listed in Table S4 in the Supplementary Material. The red colored A, B, C and F, G, H letters in parentheses define the ring and the position of the substituent in the anthrone moiety that acts as a donor or acceptor of the proton. Most of the hydrogen bond parameters shown in Table 2 and Table S4 correspond to the “peri” region of sennidin. The parameters corresponding to the bond between the COOH or CH_2_OH group and the substituents located in the “peri” region of the molecules are presented in the tables in the bold font.

The parameters listed in Table 2 and Table S4 show that the hydrogen bonds located in the “peri” region of sennidin structures are characterized by the H⋯O length of about 1.7 Å and an angle of 143–146 degrees. Other types of interactions formed between two anthrone moieties are characterized by a longer length of 1.8–2.2 Å and an angle of 148–169 degrees. The differences in the geometrical parameters of the hydrogen bonds suggest possible differences in the strength of the hydrogen bonds in the sennidin structures.

Due to the number of substituents and the possibility of O-H⋯O hydrogen bonds formation, the sennidin structures may be sensitive to the solvent interaction. Thus, the gas-phase calculations were compared with the simple PCM solvent model. The initial structures of sennidines were optimized in water and methanol environment. The results are presented in Supplementary Materials (Tables S2–S6). The A–C and F–H interplanar angles are greater for the optimized structure in solvent. However, most of the changes do not exceed 2 degrees. Only structure 14 with the O-H⋯O hydrogen bonding is an exception but the A–C and F–H interplanar angles are only 2.2 and 2.8, respectively. In addition, the geometrical parameters of the hydrogen bonds are not sensitive to the solvent. The structure 14 is again an exception with the change in the OHO angle of 18.8 degrees. Thus, the simple PCM calculation has not shown any significant difference for the structural parameters of sennidines.

Analysis of the energy and geometrical parameters indicates that the formation of the OHO hydrogen bonds linking two anthrone moieties is connected with significant, energy-consuming geometry changes, especially the changes of the interring planes. In the lowest energy structure, the hydrogen bonds between the anthrone parts are not present. For sennidin B, C and D, the energy differences between the lowest energy conformers and other conformers are not very significant, so all of the conformers can be present in plant material.

### 3.3. Analysis of Intramolecular Interactions in Sennidin Derivatives

**Figure 6:**
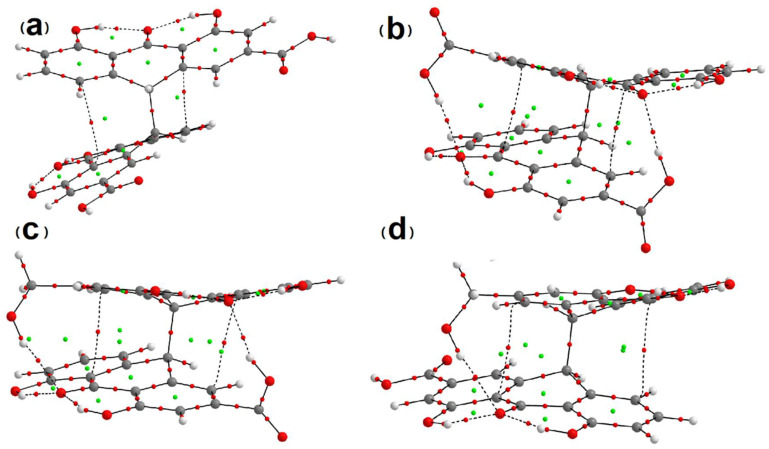
QTAIM plots for (**a**)—1, (**b**)—4, (**c**)—14, (**d**)—17 sennidin structures. Green points correspond to RCPs. Red points indicate BCPs.

As Table 2 indicates, the hydrogen bonds for the analyzed sennidin structures are not very strong. Except for the hydrogen bond, weak van der Waals interactions between both anthrone parts can be expected. In the case of very low OHO angles, the existence of the hydrogen bond can be doubtful, and a detailed analysis of the weak hydrogen bonds and van der Waals interaction must include changes in the electron density of the molecule. One of the best methods that allows examination of the electron rearrangement in the molecule is the Quantum Theory of Atoms in Molecules (QTAIM) [36] and in the case of very weak interactions—the Non-Covalent Interactions (NCI) method [33]. In terms of QTAIM, a molecule can be described as a system of critical points of electron density ρ(r). The saddle points of the electron density indicate bond-critical points (BCPs) and ring-critical points (RCPs), while the maximum of electron density represents the position of an atom and the minimum of electron density—the cage critical point. The local equation of bonding expressing the chemical action is in accordance with the Bader’s charge zero flux condition [37]. A graphical presentation of the QTAIM analysis for selected sennidin structures is presented in Figure 6. Three structures showing specific intramolecular interactions and one with no interactions have been chosen for comparison.

In the QTAIM graphs presented in Figure 6, the molecule is characterized by hydrogen bonds linking the hydroxyl groups with the central carbonyl oxygen. For structure 1, two anthrone parts are not linked by a hydrogen bond. For the other structures in Figure 6, hydrogen bonds between anthrone parts have been detected. According to the QTAIM method, the existence of a hydrogen bond is proved by an electron density path between the proton and the proton acceptor, with the presence of a bond critical point (BCP), where the gradient of electron density vanishes. A bond path is a gradient path with a BCP—a minimum electron density along the bond path and a maximum along the directions perpendicular to the bond path. Two atoms are bonded if they are located at the ends of a bond path with a BCP. A bond path except chemical bond is also common for hydrogen bonds and other interactions [38,39]. Electron density at a BCP is directly related to the interaction strength. The other parameters of electron density at a BCP quantitatively describe the interatomic interactions in the molecular system [40]. The stability of the interaction is related to ellipticity (ε) of the electron clouds at BCPs [41]. Because the bond path for stable interaction cannot be very bent, the bond path cannot be nonlinear [42]. Characteristic of the interaction presented by the bond path with a BCP is associated with characterization of the energetic properties of electron density at a BCP. The potential-energy density V(r) expresses the pressure exerted on the electrons at the BCP by the other electrons. The kinetic electronic energy G(r) at the BCP is connected with the mobility of electron density at the BCP and reflects the pressure exerted by the electrons at the BCP on the other electrons [43,44]. For the sennidin conformers presented in Figure 1, Figure 2, Figure 3 and Figure 4, all of the hydrogen bonds listed in Table S4 have been confirmed by the QTAIM parameters collated in Table S1.

**Figure 7:**
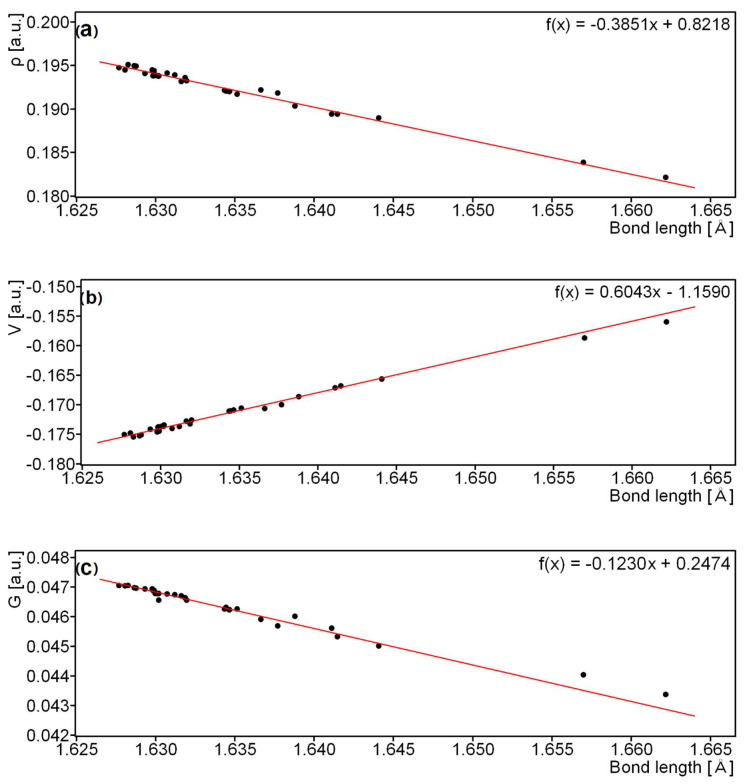
Electron density—(**a**), potential-energy density—(**b**) and kinetic-energy density—(**c**) as a function of a single C-C bond length in the sennidin structures.

In addition to the intramolecular interactions, the single elongated C-C bond which connects the anthrone moieties is very important, because the breaking of this bond results in disintegration of sennidin into two monoanthrones. Optimized structures indicate a non-standard length of the bond, which is elongated. For the investigated compounds, the bond is elongated and falls within the range of 1.625–1.665 Å, while for 1,1’,8,8’-Tetrahydroxy-10,10’-bi-9 (10H)-anthrone, its length is 1.612 Å [26]. Elongation of the C-C bond joining both parts of the dianthron occurs in the case of substitution, as in the case for Allianthrone A [28]. Figure 7 presents electron density ρ(r) and energy components V(r) and G(r) of the electrons at BCPs as a function of the bond length in Å.

The parameters of the electron density for the BCPs of the C-C bond linking the anthrone moieties of the sennidin structures show a linear dependence on the bond length and the parameters of electron density. As the length of the bond increases, the electron density at the BCP and the kinetic energy decreases, but the potential energy increases. The optimized sennidin structures indicate the elongation of the single C-C bond, which is slightly different from the standard 1.54 Å length.

### 3.4. NCI Analysis of Sennidin Derivatives

**Figure 8:**
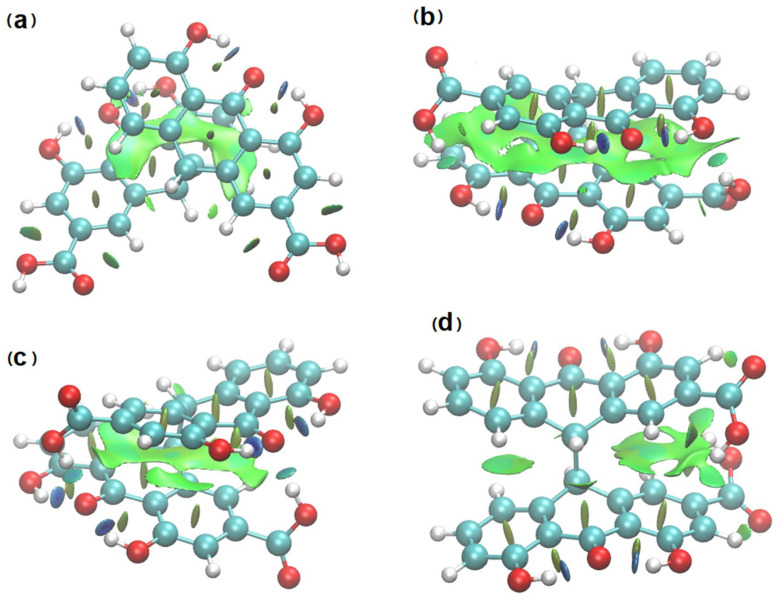
Electron density gradient isosurfaces for selected sennidin structures—1 (**a**), 3—(**b**), 4—(**c**) and 6—(**d**). The colors of the isosurfaces: blue for attractive, red for repulsive and green for intermediate-strength interactions.

The QTAIM diagram for structure 1 illustrates the compound without any hydrogen bond linking the two anthrone moieties. Despite this, elongated bond paths with a low electron density and a significant ellipticity have been found. Such bond paths are characteristic of weak, unstable interactions. For many dispersive interactions, no bond path is evident. The proper method to investigate very weak interactions is the Non-Covalent Interactions (NCI) analysis (Figure 8) [33]. This method is very convenient because it enables presentation of the interactions in the real space of the molecule. NCI analysis has been performed for the sennidin structures and NCI diagrams for selected sennidines are presented in Figure 8. The green surfaces illustrate dispersive interactions, the blue ones—hydrogen bonds.

The molecules 1 and 6 presented in Figure 8 do not contain any hydrogen bond between the anthrone moieties, but, despite the long distance between the rings, dispersive interactions are present. In the molecules with a stacking arrangement of the aromatic rings, the area of dispersive interaction spreads along the whole aromatic rings. The “peri” region of the double-anthrone moiety is characterized by the presence of strong OHO hydrogen bonds between the hydroxyl group and carbonyl oxygen. Strong interactions occur in all of the sennidin structures, which is consistent with the geometrical parameters of the bonds, presented in Table 2. The intramolecular interactions between the COOH groups and the substituents in the “peri” regions (b) and (c) can be characterized as a hydrogen bond of a medium strength. It may be noticed that the distance between two anthrone moieties in a sennidin molecule enables creation of intermediate-strength interactions, which can be described as dispersive. Rotation of the anthrone moieties changes the interactions between the two anthrone parts. As the two of the anthrone moieties stack, the dispersion increases (b). The NCI method has confirmed that the mutual rotation of the moieties of double-anthrone can influence the intramolecular interactions in the sennidin molecule. On the basis of orbital analysis, it can be assumed that the electronic structure of the sennidin can change due to the rotation around a single C-C bond.

### 3.5. Delocalization of Electrons in the Sennidin Molecule

**Figure 9:**
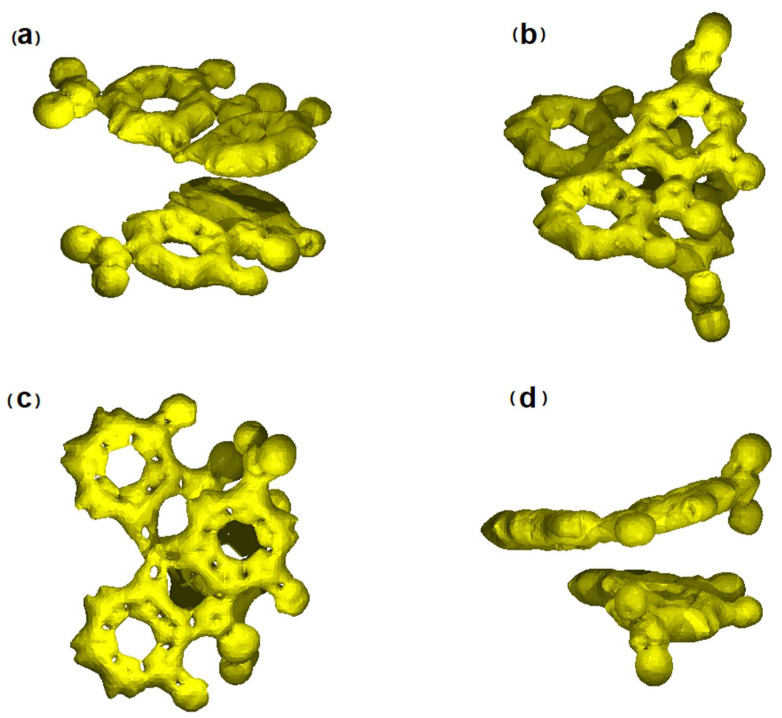
ACID isosurfaces for selected sennidin structures: (**a**) structure 1, (**b**–**d**) structure 4. ACID isosurfaces have been calculated at value of 0.05.

The distribution of electron density in the sennidin molecule can be illustrated by means of the Anisotropy of the Current-Induced Density (ACID) method [34]. Figure 9 shows graphically the ACID isosurfaces for selected sennidin structures.

**Scheme 2:**
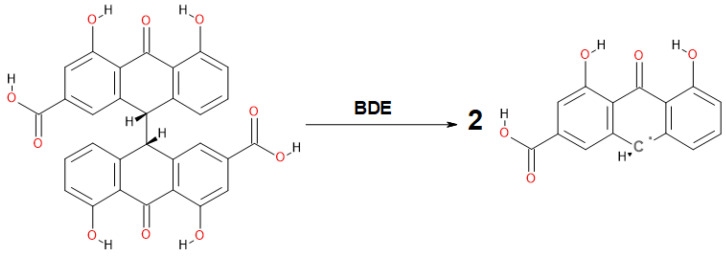
The bond dissociation energy calculation procedure for sennidin structures. (Example of sennidin A.)

Compared to the previously analyzed double-anthrone compound—hypericin^1^, the sennidin structures show delocalization only in the outer A-, C-, F-, H-rings while the B- and G-non-aromatic rings indicate localization of electron density on the carbon atoms, situated in a single C-C bond. Within the bond, there are no isosurfaces derived from the electron density π, which, according to the method, indicates the lack of induced current density. In addition, no through-space or charge-transfer interactions between the two anthrone moieties have been observed. In sum, only the outer aromatic rings and the central carbonyl group show delocalization and mobility of the charge in the sennidin molecule. The lack of current density on a single C-C bond connecting two anthrone moieties, as well as its irregular length, may explain the preferential breakdown of the sennidin molecule into monoanthrones in the gastrointestinal tract. In order to study this effect, the C-C Bond Dissociation Energy (BDE), Aromatic Interaction Energy (AIE) and Strain Energy (SE) have been calculated. The calculation of the BDE was carried in accordance with the simple radical reaction presented in Scheme 2. The AIE has been determined by the equation 1: AIE = SM + H_2_ − 2R − BDE and the SE in accordance with the equation 2: SE = SM + H_2_ − 2R, where SM corresponds to the energy of the analyzed sennidin structure, R—the radical structure of a single anthrone moiety, H_2_—energy of two hydrogen atoms, optimized with the same method as the sennidin and radical structures. All energy calculations have been performed at the temperature of 298.15 K, without zero-point-correction. The calculated energies are summarized in Supplementary Materials (Table S7).

The BDE calculated for sennidin molecules is in the range of 74–85 kcal∙mol^−1^. Although the calculated energy do not correlate with the length of the single C-C bond, the energies of the bond in the sennidin structures are lower than the standard energy in alkanes (86–88 kcal∙mol^−1^) [45]. The elongation of the bond results from the intramolecular interactions and tension in the sennidin structure. Thus, the AIE and SE have been calculated according to the modified procedure proposed by S. Grimme and C. Mück-Lichtenfeld [46]. The AIE energy structures is in the range of 25–35 kcal∙mol^−1^. The dispersive interactions demonstrated by the NCI method correlate with the AIE energy. It should be noted that in presented structures not only the aromatic interactions but also the hydrogen bonds contribute to the overall energetic effect. Such interactions induce tension in the whole molecule. On the other hand, the close position of the substituents localized in the “bay” region of the molecule produces steric effects. The SE calculated for the sennidin structures is in the range of 3–11 kcal∙mol^−1^. It can be observed that the highest SE values are found for structures in the anti-conformation with the close position of the substituents.

### 3.6. Spectroscopic Properties of Sennidines

#### 3.6.1. IR Spectra

For the best of our knowledge, there are no any good quality experimental IR spectra for the sennidines. Theoretical analysis of the sennidin structures provides information on molecular geometry, which is important when the crystal structure is not known. An additional advantage of theoretical research is the ability to predict spectroscopic properties and the shape of theoretical spectra, which can be used in a study of experimental plant material.

**Figure 10:**
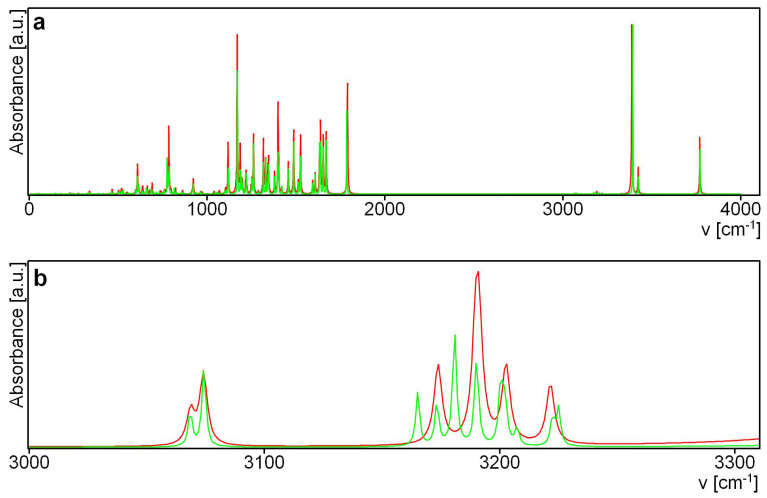
Theoretical IR spectra for sennidin (**a**) and sennidin (**b**). Structure 1—red, structure 7—green.

Figure 10 compares the theoretical IR spectra for sennidin structures 1 and 7. Because the structure of both sennidines is very similar, their spectra are identical, except for the very low intensity bands of about 3100 cm^−1^. The band at 3174 cm^−1^ for structure 1 corresponds to the stretching C–H vibrations of both aromatic rings without a carboxylic group. For structure 7, this band is shifted to 3164 cm^−1^ and two new C–H stretching bands arise each for an aromatic ring unsubstituted with a carboxylic group.

When the IR spectra of structures 1 and 7 are almost identical, the differences in the spectra for 11 and 17 are more significant. The spectra obtained for sennidin C and D (structure 11 and 17) are presented in Supplementary Material (Figure S1). The first band which arises in the IR spectrum of 11, but not of 17, at 3858 cm^−1^, is the stretching of OH for the CH_2_OH group. In the spectrum of 17, this band is shifted up to 3768 cm^−1^. The stretching of the OH groups linked by the hydrogen bond to carbonyl oxygen are located at 3374 cm^−1^ for 11 and 3365 cm^−1^ for 17. In the range of the stretching of aromatic C–H vibrations, the bands are located at 3189, 3175 and 3148 cm^−1^, while for 17—at 3180 and 3164 cm^−1^. The stretching for the C–H bonds to the central rings of both parts of anthrone is at 3072 cm^−1^ and 3063 cm^−1^ for 11, and 3072 and 3075 cm^−1^ for 17. In addition, the CH stretching vibrations of the CH_2_OH group are shifted from 2997 cm^−1^ and 2970 cm^−1^ for 11 to 3062 cm^−1^ and 3003 cm^−1^ for 17. In the range typical for out-of-plane OH vibrations, characteristic bands for 11 are located at 797 and 780 cm^−1^ and for 17—at 788, 769, 764, 487 and 473 cm^−1^. In the far IR region, there are intensive out-of-plane bands for a free OH bond for 11 (242, 232, 230, 226, 193 cm^−1^) but analogous bands in the spectrum of 17 have not been detected.

To check if the differences in the IR spectrum can be analyzed for structures with a similar energy, in Figure S2 (Supplementary Material) the spectra for 7, 8, 9, 10 are compared. For 9, a separate band at 3731 cm^−1^ is ascribed to the OH stretching vibrations of the carboxylic group participating in the OHO hydrogen bond linking two monoanthrone parts. At around 3400 cm^−1^, stretching vibrations for double OH⋯O to carbonyl for all of the analyzed structures are located, but in **9** those bands are shifted compared with the other structures.

In the region of stretching of carbonyl C=O groups, two bands for two COOH groups are present. As regards structures 7 and 8, both carboxyl groups are free and those bands are located at 1786 and 1784 cm^−1^ for 7 and at 1795 and 1785 cm^−1^ for 8. In the case of 9 and 10, one of the carboxylic group is connected to the oxygen of another monoanthron and, depending on the hydrogen bond strength, these bands are shifted to 1830 and 1786 cm^−1^ in 9 and 1818 and 1788 cm^−1^ in 10.

The range below 1670 cm^−1^ is typical of in-plane ring-bending vibrations. The IR spectrum is similar for the analyzed derivatives, except for 9, in the case of which the bending vibration bands are shifted in comparison with the bands in the other structures. A detailed analysis of the theoretical IR spectra might allow details characteristic of a particular structure to be established, which might be useful for analyzing experimental plant material.

#### 3.6.2. UV Spectra

**Figure 11:**
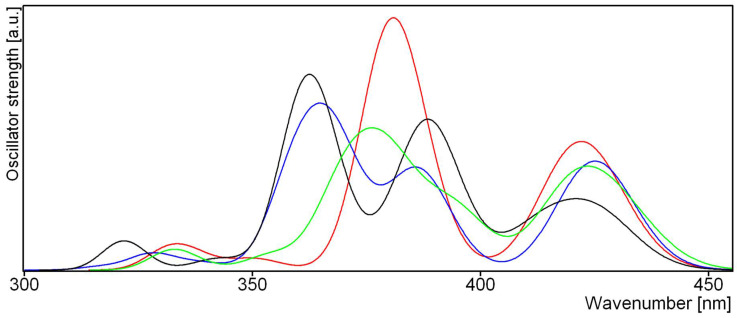
Theoretical excitation spectra for sennidin derivatives 1—red, 7—green, 11—blue and 17—black.

QTAIM analysis of electron density can be complemented with a traditional description of the molecular orbitals. To investigate whether the mutual rotation of the anthrone moieties affects the electronic structure of sennidin, analysis of the molecular orbitals and excitation spectra was performed for sennidin derivatives 1, 7, 11 and 17. Figure 11 presents the excitation spectra for the analyzed sennidin derivatives.

Conformational changes of sennidines are reflected in the electron density and excitation spectra for the allowed electron transition. The spectra are presented in Figure 11 and the molecular orbitals which participate in the singlet–singlet transitions (Figures S2–S6) and their parameters (Table S8) are presented in the Supplementary Materials.

The sensitivity of the molecular orbitals to intramolecular interaction connected to the rotation of the anthrone moieties in sennidin derivatives is reflected in excitation spectra. Using excitation spectra, it is not only easy to distinguish sennidines with two carboxylate groups from those with a carboxylate and methoxy group, but also to see the differences between A and B or C and D derivatives.

## 4. Conclusions

Many optimized sennidin structures are presented in the article to illustrate the possible relationship between the energy of the conformers and the arrangement of the anthrone moieties as well as the intramolecular interactions. According to the gas-phase optimization and to the solvent PCM model for sennidines, many conformations that differ in spatial arrangement and intramolecular interactions are present; however, their energy does not exceed 2 kcal∙mol^−1^ from the minimum. The lowest energy structure that is most likely to be found in plant material is characterized by the presence of OHO hydrogen bonds formed by hydroxyl groups and carbonyl oxygen, but formation of hydrogen bonds between anthrone substituents is associated with a higher energy used to change the geometry of both anthrone parts, although it can be assumed that the formation of intramolecular interactions reduce the energy of the system. Stacking conformation of the anthrone parts of sennidin increases the dispersive interactions so the overall energetic effect of the molecule is ambiguous. However, for some sennidin derivatives, the energy difference between particular structures is very low and stacking structures with hydrogen bonds linking the anthrone parts may exist in plant material. Spectroscopic analysis performed for theoretical structures can be useful in studying the structure of the experimental plant material.

It can be assumed that sennidines can occur naturally in the form of the lowest energy conformers. In silico studies performed in this work suggests the presence of an extended C-C bond, linking the anthrone moieties. Owing to this, the mechanism of the preferential breakdown of sennidines into monoanthrones, which is a key aspect of the pharmacological properties of these compounds, was elucidated. The analysis of the available literature and the use of solid and proven theoretical methods contribute to the further development of research on dianthrones of natural origin.

## Figures and Tables

**Table 1:** Angles between the ring planes in sennidin conformers. “A”—unsubstituted double anthrone in crystal and “B”—optimized double-anthrone. σ = 0.001 [deg.].

Compound	Structure	Plane/Angle [Deg.]	
		A–C	F–H
Sennidin A	1	17.700	17.701
	2	22.040	20.086
	3	11.944	11.913
	4	18.993	18.990
	5	20.256	22.294
	6	25.014	25.017
Sennidin B	7	16.229	17.101
	8	14.540	16.816
	9	11.855	18.733
	10	18.915	16.502
Sennidin C	11	17.479	16.784
	12	22.503	26.483
	13	14.519	11.452
	14	13.671	14.046
	15	23.246	26.664
	16	25.190	23.316
Sennidin D	17	18.724	14.341
	18	16.381	17.255
	19	20.767	14.317
	20	20.403	15.657
	21	23.629	24.659
A (Figure 5)	-	17.302	17.309
B (Figure 5)	-	11.947	11.947

**Table 2:** Hydrogen bonds in sennidin A–D lowest energy structures, crystal “A” and calculated “B” double-anthrone moieties, σ = 0.0001 [Å], σ = 0.001 [deg.].

Compound	Structure	Hydrogen Bond	H⋯O [Å]	O⋯O [Å]	OHO [Deg.]
Sennidin A	1	C(A)-O-H⋯O=C(B)	1.7142	2.5852	145.43
		C(C)-O-H⋯O=C(B)	1.706	2.5813	145.897
		C(F)-O-H⋯O=C(G)	1.7142	2.5852	145.43
		C(H)-O-H⋯O=C(G)	1.7061	2.5813	145.897
Sennidin B	7	C(A)-O-H⋯O=C(B)	1.7096	2.5841	145.868
		C(C)-O-H⋯O=C(B)	1.7142	2.5856	145.446
		C(F)-O-H⋯O=C(G)	1.7135	2.585	145.494
		C(H)-O-H⋯O=C(G)	1.7068	2.5818	145.919
Sennidin C	11	C(A)-O-H⋯O=C(B)	1.715	2.5859	145.44
		C(C)-O-H⋯O=C(B)	1.7036	2.5801	146.018
		C(F)-O-H⋯O=C(G)	1.7043	2.5798	145.92
		C(H)-O-H⋯O=C(G)	1.705	2.5831	146.3
Sennidin D	17	C(A)-O-H⋯O=C(B)	1.7156	2.5887	145.805
		C(C)-O-H⋯O=C(B)	1.7478	2.6091	144.62
		C(F)-O-H⋯O=C(G)	1.7001	2.578	146.235
		C(H)-O-H⋯O=C(G)	1.6976	2.5748	146.066
		C(H)-C(H_2_)-O-H⋯O=C(B)	2.1931	3.0922	154.333
A	-	C(A)-O-H⋯O=C(B)	1.6862	2.5831	151.734
		C(C)-O-H⋯O=C(B)	1.7383	2.5646	146.429
		C(F)-O-H⋯O=C(G)	1.7383	2.5646	146.429
		C(H)-O-H⋯O=C(G)	1.6862	2.5831	151.734
B	-	C(A)-O-H⋯O=C(B)	1.7001	2.5783	146.268
		C(C)-O-H⋯O=C(B)	1.7077	2.5814	145.74
		C(F)-O-H⋯O=C(G)	1.7077	2.5815	145.737
		C(H)-O-H⋯O=C(G)	1.7002	2.5784	146.266

## Data Availability

The data presented in this study are available in supplementary material. Xyz or wfn files are available on request from the corresponding author.

## Supplementary Materials

The following are available online at https://www.mdpi.com/article/10.3390/biology10060468/s1, Table S1. Electron Density ρ(r) and Energy Components V(r), G(r) at RCPs for Sennidin Structures, σ = 0.00001 [a.u.], Table S2. Angles Between the Ring Planes in Sennidin Conformers—Water, σ = 0.001 [deg.], Table S3. Angles Between the Ring Planes in Sennidin Conformers—Methanol, σ = 0.001 [deg.], Table S4. Angles Between the Ring Planes in Sennidin Conformers. “A”—unsubstituted double anthrone in crystal and “B”—optimized double-anthrone. σ = 0.001 [deg.]. σ = 0.0001 [Å], σ = 0.001 [deg.], Table S5. Hydrogen Bonds in Sennidin Conformers. PCM optimization in Water, σ = 0.0001 [Å], σ = 0.001 [deg.], Table S6. Hydrogen Bonds in Sennidin Conformers. PCM optimization in Methanol, σ = 0.0001 [Å], σ = 0.001 [deg.], Table S7. The C-C Bond Length, BDE, AIE and SE Calculated for Sennidin Structures, Table S8. Orbital Transition Parameters for Sennidin Derivatives, Figure S1. Theoretical IR Spectra for Sennidine C and Sennidine D. Structure 11—Red, Structure 17—Green, Figure S2. Theoretical IR Spectra for Sennidine B. Structure 7—Blue, 8—Black, 9—Red, 10—Green, Figure S3. Molecular Orbitals for Sennidine A—Structure 1, Figure S4. Molecular Orbitals for Sennidine B—Structure 7, Figure S5. Molecular Orbitals for Sennidine C—Structure 11, Figure S6. Molecular Orbitals for Sennidine D—Structure 17.
