# Supplementary material for: In Silico Studies on Sennidines—Natural Dianthrones from Senna
Source: Biology (Basel). 2021 May 26;10(6):468. doi: 10.3390/biology10060468 (PMC8227258; doi:10.3390/biology10060468)
Supplement: Supplementary file 1 [file biology-10-00468-s001.zip › biology-1202874 supplementary.pdf]

biology

MDPI

SUPPORTING INFORMATION

Table S1. Electron Density ϱ(r) and Energy Components V(r), G(r) at RCPs for Sennidin Structures, σ=0.00001 [a.u.].

Conformer	Ring	ϱ(r) [a.u.]	V(r) [a.u.]	G(r) [a.u.]
**1**	A	0.02130	−0.02390	0.03109
	B	0.01855	−0.01917	0.02531
	C	0.02139	−0.02393	0.03114
	F	0.02130	−0.02390	0.03109
	G	0.01855	−0.01917	0.02531
	H	0.02139	−0.02393	0.03114
**2**	A	0.02116	−0.02375	0.03103
	B	0.01893	−0.01977	0.02599
	C	0.02149	−0.02404	0.03122
	F	0.02115	−0.02373	0.03101
	G	0.01889	−0.01970	0.02593
	H	0.02150	−0.02406	0.03125
**3**	A	0.02148	−0.02416	0.03139
	B	0.01877	−0.01958	0.02579
	C	0.02153	−0.02413	0.03127
	F	0.02148	−0.02416	0.03139
	G	0.01877	−0.01958	0.02579
	H	0.02153	−0.02413	0.03127
**4**	A	0.02118	−0.02378	0.03107
	B	0.01884	−0.01965	0.02588
	C	0.02131	−0.02384	0.03099
	F	0.02118	−0.02378	0.03107
	G	0.01884	−0.01966	0.02588
	H	0.02131	−0.02384	0.03099
**5**	A	0.02116	−0.02374	0.03101
	B	0.01891	−0.01974	0.02598
	C	0.02146	−0.02401	0.03119
	F	0.02118	−0.02378	0.03104
	G	0.01897	−0.01982	0.02605
	H	0.02150	−0.02407	0.03124
**6**	A	0.02122	−0.02381	0.03107
	B	0.01898	−0.01981	0.02604
	C	0.02146	−0.02401	0.03115
	F	0.02122	−0.02381	0.03107
	G	0.01898	−0.01981	0.02604
	H	0.02146	−0.02401	0.03115
**7**	A	0.02127	−0.02387	0.03112
	B	0.01855	−0.01917	0.02532
	C	0.02145	−0.02398	0.03114
	F	0.02131	−0.02391	0.03110
	G	0.01855	−0.01919	0.02533

	H	0.02140	−0.02394	0.03114
**8**	A	0.02125	−0.02385	0.03111
	B	0.01848	−0.01907	0.02521
	C	0.02143	−0.02394	0.03110
	F	0.02129	−0.02388	0.03107
	G	0.01851	−0.01914	0.02528
	H	0.02139	−0.02393	0.03114
**9**	A	0.02123	−0.02383	0.03109
	B	0.01849	−0.01912	0.02528
	C	0.02145	−0.02406	0.03124
	F	0.02129	−0.02388	0.03106
	G	0.01865	−0.01933	0.02549
	H	0.02135	−0.02387	0.03108
**10**	A	0.02130	−0.02390	0.03114
	B	0.01864	−0.01931	0.02547
	C	0.02143	−0.02393	0.03108
	F	0.02130	−0.02390	0.03109
	G	0.01854	−0.01917	0.02532
	H	0.02149	−0.02406	0.03120
**11**	A	0.02130	−0.02389	0.03109
	B	0.01854	−0.01915	0.02530
	C	0.02139	−0.02393	0.03114
	F	0.02131	−0.02392	0.03112
	G	0.01854	−0.01918	0.02534
	H	0.02135	−0.02392	0.03113
**12**	A	0.02124	−0.02383	0.03108
	B	0.01894	−0.01975	0.02598
	C	0.02143	−0.02398	0.03119
	F	0.02128	−0.02389	0.03114
	G	0.01903	−0.01989	0.02611
	H	0.02138	−0.02394	0.03111
**13**	A	0.02145	−0.02412	0.03135
	B	0.01885	−0.01970	0.02592
	C	0.02153	−0.02413	0.03128
	F	0.02150	−0.02419	0.03143
	G	0.01870	−0.01948	0.02567
	H	0.02148	−0.02407	0.03119
**14**	A	0.02122	−0.02382	0.03108
	B	0.01859	−0.01928	0.02548
	C	0.02139	−0.02398	0.03114
	F	0.02120	−0.02379	0.03105
	G	0.01866	−0.01939	0.02559
	H	0.02141	−0.02397	0.03111
**15**	A	0.02125	−0.02385	0.03110
	B	0.01900	−0.01985	0.02608
	C	0.02147	−0.02405	0.03126

	F	0.02126	−0.02386	0.03111
	G	0.01900	−0.01984	0.02606
	H	0.02132	−0.02387	0.03106
**16**	A	0.02121	−0.02381	0.03107
	B	0.01893	−0.01975	0.02598
	C	0.02140	−0.02392	0.03110
	F	0.02123	−0.02383	0.03108
	G	0.01902	−0.01990	0.02613
	H	0.02151	−0.02414	0.03127
**17**	A	0.02129	−0.02386	0.03105
	B	0.01861	−0.01927	0.02544
	C	0.02132	−0.02385	0.03109
	F	0.02123	−0.02384	0.03111
	G	0.01844	−0.01903	0.02518
	H	0.02135	−0.02391	0.03106
**18**	A	0.02132	−0.02392	0.03112
	B	0.01867	−0.01938	0.02556
	C	0.02156	−0.02416	0.03135
	F	0.02130	−0.02391	0.03115
	G	0.01873	−0.01948	0.02565
	H	0.02146	−0.02405	0.03119
**19**	A	0.02130	−0.02387	0.03110
	B	0.01890	−0.01972	0.02591
	C	0.02152	−0.02407	0.03123
	F	0.02138	−0.02401	0.03120
	G	0.01865	−0.01940	0.02557
	H	0.02147	−0.02402	0.03119
**20**	A	0.02131	−0.02390	0.03114
	B	0.01873	−0.01945	0.02563
	C	0.02145	−0.02398	0.03114
	F	0.02134	−0.02395	0.03115
	G	0.01857	−0.01925	0.02542
	H	0.02148	−0.02406	0.03118
**21**	A	0.02128	−0.02388	0.03112
	B	0.01896	−0.01979	0.02602
	C	0.02137	−0.02387	0.03108
	F	0.02132	−0.02394	0.03115
	G	0.01902	−0.01988	0.02610
	H	0.02139	−0.02395	0.03114

Table S2. Angles Between the Ring Planes in Sennidin Conformers – Water, σ=0.001 [deg.].

**Compound**	**Conformer**	**Plane/angle [deg.]**	
		**A–C**	**F–H**
Sennidin A	1	18.367	18.358
	2	22.116	20.003

	3	12.904	12.910
	4	19.249	19.245
	5	20.339	21.971
	6	26.226	26.221
Sennidin B	7	16.741	17.930
	8	16.255	17.577
	9	12.679	19.451
	10	18.510	17.327
Sennidin C	11	18.414	17.548
	12	22.902	27.256
	13	15.032	13.049
	14	15.869	16.824
	15	23.315	27.079
	16	25.734	24.315
Sennidin D	17	19.435	15.321
	18	17.049	18.180
	19	21.136	14.912
	20	19.605	15.500
	21	24.575	24.725

Table S3. Angles Between the Ring Planes in Sennidin Conformers – Methanol, σ=0.001 [deg.].

**Compound**	**Conformer**	**Plane/angle [deg.]**	
		**A–C**	**F–H**
Sennidin A	1	18.330	18.321
	2	22.135	19.997
	3	12.867	12.868
	4	19.287	19.281
	5	20.327	22.085
	6	26.106	26.106
Sennidin B	7	16.849	17.976
	8	16.192	17.523
	9	12.623	19.440
	10	18.580	17.305
Sennidin C	11	18.379	17.512
	12	22.911	27.215
	13	14.943	12.917
	14	15.901	16.808
	15	23.331	27.076
	16	25.722	24.286
Sennidin D	17	19.430	15.294
	18	17.027	18.159
	19	21.119	14.918
	20	19.566	15.541
	21	24.551	24.761

Table S4. Angles Between the Ring Planes in Sennidin Conformers. "A" – unsubstituted double anthrone in crystal and "B" – optimized double–anthrone. σ=0.001 [deg.]. σ=0.0001 [Å], σ=0.001 [deg.].

Compound	Structure	Hydrogen bond	H···O [Å]	O···O [Å]	OHO [deg.]
sennidin A	1	C(A)–O–H···O=C(B)	1.7142	2.5852	145.430
		C(C)–O–H···O=C(B)	1.7060	2.5813	145.897
		C(F)–O–H···O=C(G)	1.7142	2.5852	145.430
		C(H)–O–H···O=C(G)	1.7061	2.5813	145.897
	2	C(A)–O–H···O=C(B)	1.7266	2.5969	145.362
		C(C)–O–H···O=C(B)	1.7294	2.6000	145.338
		C(F)–O–H···O=C(G)	1.7272	2.5964	145.214
		C(H)–O–H···O=C(G)	1.7311	2.6001	145.155
		**C(C)–C(O)–OH···O(H)–C(O)–C(H)**	**2.0086**	**2.9479**	**161.947**
	3	C(A)–O–H···O=C(B)	1.6827	2.5682	146.877
		C(C)–O–H···O=C(B)	1.7273	2.5958	145.140
		C(F)–O–H···O=C(G)	1.6826	2.5681	146.880
		C(H)–O–H···O=C(G)	1.7273	2.5958	145.135
		**C(C)–C(O)–OH···O(H)–C(F)**	**2.1723**	**3.0426**	**148.385**
		**C(H)–C(O)–OH···O(H)–C(A)**	**2.1727**	**3.0427**	**148.347**
	4	C(A)–O–H···O=C(B)	1.7691	2.6223	143.635
		C(C)–O–H···O=C(B)	1.7303	2.5972	145.052
		C(F)–O–H···O=C(G)	1.7691	2.6223	143.637
		C(H)–O–H···O=C(G)	1.7303	2.5971	145.049
		C(C)–C(O)–OH···O=C(G)	1.8972	2.8674	172.762
		C(H)–C(O)–OH···O=C(B)	1.8976	2.8678	172.761
	5	C(A)–O–H···O=C(B)	1.7315	2.5989	145.015
		C(C)–O–H···O=C(B)	1.7275	2.5971	145.167
		C(F)–O–H···O=C(G)	1.7256	2.5966	145.457
		C(H)–O–H···O=C(G)	1.7311	2.6021	145.479
		**C(H)–C(O)–OH···O=C(OH)C(C)**	**1.8381**	**2.8053**	**168.771**
	6	C(A)–O–H···O=C(B)	1.7362	2.6026	144.925
		C(C)–O–H···O=C(B)	1.7288	2.5982	145.171
		C(F)–O–H···O=C(G)	1.7362	2.6026	144.926
		C(H)–O–H···O=C(G)	1.7288	2.5982	145.171
sennidin B	7	C(A)–O–H···O=C(B)	1.7096	2.5841	145.868
		C(C)–O–H···O=C(B)	1.7142	2.5856	145.446
		C(F)–O–H···O=C(G)	1.7135	2.5850	145.494
		C(H)–O–H···O=C(G)	1.7068	2.5818	145.919
	8	C(A)–O–H···O=C(B)	1.7071	2.5818	145.872
		C(C)–O–H···O=C(B)	1.7100	2.5820	145.528
		C(F)–O–H···O=C(G)	1.7149	2.5854	145.347
		C(H)–O–H···O=C(G)	1.7074	2.5821	145.806
	9	C(A)–O–H···O=C(B)	1.7085	2.5829	145.844
		C(C)–O–H···O=C(B)	1.7022	2.5778	145.823
		C(F)–O–H···O=C(G)	1.7214	2.5930	145.734
		C(H)–O–H···O=C(G)	1.7527	2.6128	144.442
	10	C(A)–O–H···O=C(B)	1.7157	2.5884	145.649
		C(C)–O–H···O=C(B)	1.7227	2.5917	145.187
		C(F)–O–H···O=C(G)	1.7236	2.5903	144.921
		C(H)–O–H···O=C(G)	1.7062	2.5791	145.508
sennidin C	11	C(A)–O–H···O=C(B)	1.7150	2.5859	145.440
		C(C)–O–H···O=C(B)	1.7036	2.5801	146.018
		C(F)–O–H···O=C(G)	1.7043	2.5798	145.920

		C(H)–O–H···O=C(G)	1.7050	2.5831	146.300
	12	C(A)–O–H···O=C(B)	1.7268	2.5970	145.393
		C(C)–O–H···O=C(B)	1.7232	2.5946	145.449
		C(F)–O–H···O=C(G)	1.7225	2.5962	145.766
		C(H)–O–H···O=C(G)	1.7265	2.5988	145.599
		**C(H)–C(H_2)–O–H···O(H)–C(O)–C(C)**	**2.2492**	**3.2019**	**169.144**
	13	C(A)–O–H···O=C(B)	1.6913	2.5749	146.636
		C(C)–O–H···O=C(B)	1.7371	2.6041	145.005
		C(F)–O–H···O=C(G)	1.6578	2.5514	147.698
		C(H)–O–H···O=C(G)	1.7269	2.5954	145.280
		**C(C)–C(O)–O–H···O(H)–C(F)**	**2.0939**	**2.9798**	**150.585**
		**C(H)–C(H_2)–O–H···O(H)–C(A)**	**2.1529**	**3.1070**	**168.394**
	14	C(A)–O–H···O=C(B)	1.7316	2.5982	145.145
		C(C)–O–H···O=C(B)	1.7207	2.5915	145.507
		C(F)–O–H···O=C(G)	1.7521	2.6126	144.518
		C(H)–O–H···O=C(G)	1.7212	2.5929	145.794
		**C(C)–C(O)–O–H···O=C(G)**	**2.0046**	**2.9108**	**153.912**
		**C(H)–C(H_2)–O–H···O=C(B)**	**2.1301**	**3.0741**	**164.956**
	15	C(A)–O–H···O=C(B)	1.7240	2.5962	145.633
		C(C)–O–H···O=C(B)	1.7221	2.5961	145.804
		C(F)–O–H···O=C(G)	1.7268	2.5981	145.495
		C(H)–O–H···O=C(G)	1.7268	2.5976	145.357
		**C(C)–C(O)–O–H···O=C(H)–C(H_2)–C(H)**	**1.8108**	**2.7717**	**165.578**
	16	C(A)–O–H···O=C(B)	1.7315	2.5997	145.130
		C(C)–O–H···O=C(B)	1.7269	2.5968	145.244
		C(F)–O–H···O=C(G)	1.7281	2.5987	145.411
		C(H)–O–H···O=C(G)	1.7276	2.6004	145.617
sennidin D	17	C(A)–O–H···O=C(B)	1.7156	2.5887	145.805
		C(C)–O–H···O=C(B)	1.7478	2.6091	144.620
		C(F)–O–H···O=C(G)	1.7001	2.5780	146.235
		C(H)–O–H···O=C(G)	1.6976	2.5748	146.066
		**C(H)–C(H_2)–O–H···O=C(B)**	**2.1931**	**3.0922**	**154.333**
	18	C(A)–O–H···O=C(B)	1.7291	2.5941	144.766
		C(C)–O–H···O=C(B)	1.6687	2.5577	147.168
		C(F)–O–H···O=C(G)	1.7074	2.5844	146.132
		C(H)–O–H···O=C(G)	1.7271	2.5982	145.578
		**C(H)–C(H_2)–O–H···O(H)–C(C)**	**2.1320**	**3.0885**	**169.722**
	19	C(A)–O–H···O=C(B)	1.7219	2.5935	145.525
		C(C)–O–H···O=C(B)	1.7344	2.6026	145.142
		C(F)–O–H···O=C(G)	1.7095	2.5833	145.719
		C(H)–O–H···O=C(G)	1.7007	2.5782	146.191
		**C(H)–C(H_2)–O–H···O=C(OH)–C(C)**	**2.0545**	**3.0061**	**166.977**
	20	C(A)–O–H···O=C(B)	1.7169	2.5896	145.628
		C(C)–O–H···O=C(B)	1.7314	2.5990	145.068
		C(F)–O–H···O=C(G)	1.7195	2.5884	145.166
		C(H)–O–H···O=C(G)	1.6986	2.5755	145.955
	21	C(A)–O–H···O=C(B)	1.7298	2.5990	145.252
		C(C)–O–H···O=C(B)	1.7262	2.5968	145.383
		C(F)–O–H···O=C(G)	1.7239	2.5967	145.690
		C(H)–O–H···O=C(G)	1.7263	2.5998	145.758
A (Figure 5)	–	C(A)–O–H···O=C(B)	1.6862	2.5831	151.734
		C(C)–O–H···O=C(B)	1.7383	2.5646	146.429
		C(F)–O–H···O=C(G)	1.7383	2.5646	146.429
		C(H)–O–H···O=C(G)	1.6862	2.5831	151.734

B (Figure 5)	–	C(A)–O–H···O=C(B)	1.7001	2.5783	146.268
		C(C)–O–H···O=C(B)	1.7077	2.5814	145.740
		C(F)–O–H···O=C(G)	1.7077	2.5815	145.737
		C(H)–O–H···O=C(G)	1.7002	2.5784	146.266

Table S5. Hydrogen Bonds in Sennidin Conformers. PCM optimization in Water, σ=0.0001 [Å], σ=0.001 [deg.].

Compound	Conformer	Hydrogen bond	H···O [Å]	O···O [Å]	OHO [deg.]
sennidin A	1	C(A)–O–H···O=C(B)	1.7035	2.5805	146.115
		C(C)–O–H···O=C(B)	1.7030	2.5810	146.223
		C(F)–O–H···O=C(G)	1.7036	2.5806	146.117
		C(H)–O–H···O=C(G)	1.7030	2.5810	146.224
	2	C(A)–O–H···O=C(B)	1.7206	2.5947	145.845
		C(C)–O–H···O=C(B)	1.7142	2.5916	146.107
		C(F)–O–H···O=C(G)	1.7182	2.5925	145.849
		C(H)–O–H···O=C(G)	1.7150	2.5918	146.053
		**C(C)–C(O)–OH···O(H)–C(O)–C(H)**	**1.9498**	**2.8931**	**161.954**
	3	C(A)–O–H···O=C(B)	1.6904	2.5734	146.576
		C(C)–O–H···O=C(B)	1.7124	2.5877	145.878
		C(F)–O–H···O=C(G)	1.6908	2.5738	146.571
		C(H)–O–H···O=C(G)	1.7124	2.5877	145.875
		**C(C)–C(O)–OH···O(H)–C(F)**	**2.1446**	**3.0113**	**147.422**
		**C(H)–C(O)–OH···O(H)–C(A)**	**2.1445**	**3.0115**	**147.461**
	4	C(A)–O–H···O=C(B)	1.7626	2.6202	144.157
		C(C)–O–H···O=C(B)	1.7186	2.5903	145.487
		C(F)–O–H···O=C(G)	1.7627	2.6204	144.155
		C(H)–O–H···O=C(G)	1.7186	2.5904	145.487
		**C(C)–C(O)–OH···O=C(G)**	**1.8451**	**2.8208**	**173.359**
		**C(H)–C(O)–OH···O=C(B)**	**1.8453**	**2.8210**	**173.351**
	5	C(A)–O–H···O=C(B)	1.7213	2.5945	145.731
		C(C)–O–H···O=C(B)	1.7133	2.5904	146.058
		C(F)–O–H···O=C(G)	1.7197	2.5942	145.887
		C(H)–O–H···O=C(G)	1.7145	2.5919	145.123
		**C(H)–C(O)–OH···O=C(OH)C(C)**	**1.7680**	**2.7456**	**170.459**
	6	C(A)–O–H···O=C(B)	1.7267	2.5984	145.561
		C(C)–O–H···O=C(B)	1.7170	2.5922	145.815
		C(F)–O–H···O=C(G)	1.7267	2.5985	145.562
		C(H)–O–H···O=C(G)	1.7170	2.5922	145.816
sennidin B	7	C(A)–O–H···O=C(B)	1.7070	2.5840	146.198
		C(C)–O–H···O=C(B)	1.6989	2.5771	146.175
		C(F)–O–H···O=C(G)	1.7051	2.5817	146.077
		C(H)–O–H···O=C(G)	1.7016	2.5799	146.247
	8	C(A)–O–H···O=C(B)	1.7064	2.5835	146.201
		C(C)–O–H···O=C(B)	1.6987	2.5769	146.184
		C(F)–O–H···O=C(G)	1.7040	2.5808	146.093
		C(H)–O–H···O=C(G)	1.7012	2.5795	146.251
	9	C(A)–O–H···O=C(B)	1.7052	2.5824	146.211
		C(C)–O–H···O=C(B)	1.6871	2.5691	146.491
		C(F)–O–H···O=C(G)	1.7195	2.5922	145.717
		C(H)–O–H···O=C(G)	1.7413	2.6075	145.083
	10	C(A)–O–H···O=C(B)	1.7131	2.5881	145.975

		C(C)–O–H···O=C(B)	1.7039	2.5808	146.003
		C(F)–O–H···O=C(G)	1.7099	2.5840	145.797
		C(H)–O–H···O=C(G)	1.7005	2.5782	146.118
sennidin C	11	C(A)–O–H···O=C(B)	1.7045	2.5814	146.127
		C(C)–O–H···O=C(B)	1.7017	2.5803	146.293
		C(F)–O–H···O=C(G)	1.6908	2.5735	146.761
		C(H)–O–H···O=C(G)	1.6986	2.5808	146.793
	12	C(A)–O–H···O=C(B)	1.7195	2.5940	145.894
		C(C)–O–H···O=C(B)	1.7130	2.5898	146.080
		C(F)–O–H···O=C(G)	1.7147	2.5929	146.332
		C(H)–O–H···O=C(G)	1.7157	2.5939	146.288
		**C(H)–C(H_2)–O–H···O(H)–C(O)–C(C)**	**2.1544**	**3.1183**	**175.022**
	13	C(A)–O–H···O=C(B)	1.6947	2.5784	146.757
		C(C)–O–H···O=C(B)	1.7165	2.5922	145.954
		C(F)–O–H···O=C(G)	1.6658	2.5576	147.475
		C(H)–O–H···O=C(G)	1.7109	2.5873	146.172
		**C(C)–C(O)–O–H···O(H)–C(F)**	**2.0501**	**2.9476**	**152.042**
		**C(H)–C(H_2)–O–H···O(H)–C(A)**	**2.1178**	**3.0801**	**171.959**
	14	C(A)–O–H···O=C(B)	1.7228	2.5934	145.572
		C(C)–O–H···O=C(B)	1.6983	2.5754	146.078
		C(F)–O–H···O=C(G)	1.7621	2.6193	144.115
		C(H)–O–H···O=C(G)	1.7206	2.5943	145.886
		**C(C)–C(O)–O–H···O=C(G)**	**1.8372**	**2.8131**	**172.670**
		**C(H)–C(H_2)–O–H···O=C(B)**	**2.0332**	**2.9941**	**171.066**
	15	C(A)–O–H···O=C(B)	1.7171	2.5930	146.063
		C(C)–O–H···O=C(B)	1.7121	2.5906	146.221
		C(F)–O–H···O=C(G)	1.7163	2.5935	146.214
		C(H)–O–H···O=C(G)	1.7163	2.5936	146.159
		**C(C)–C(O)–O–H···O=C(H)–C(H_2)–C(H)**	**1.6937**	**2.6806**	**171.045**
	16	C(A)–O–H···O=C(B)	1.7243	2.5969	145.672
		C(C)–O–H···O=C(B)	1.7146	2.5908	145.959
		C(F)–O–H···O=C(G)	1.7169	2.5935	146.138
		C(H)–O–H···O=C(G)	1.7163	2.5949	146.310
sennidin D	17	C(A)–O–H···O=C(B)	1.7067	2.5835	146.124
		C(C)–O–H···O=C(B)	1.7356	2.6020	145.084
		C(F)–O–H···O=C(G)	1.6932	2.5750	146.719
		C(H)–O–H···O=C(G)	1.6847	2.5679	146.721
		**C(H)–C(H_2)–O–H···O=C(B)**	**2.2266**	**3.0987**	**149.515**
	18	C(A)–O–H···O=C(B)	1.7138	2.5870	145.722
		C(C)–O–H···O=C(B)	1.6747	2.5625	147.082
		C(F)–O–H···O=C(G)	1.7024	2.5825	146.562
		C(H)–O–H···O=C(G)	1.7075	2.5866	146.461
		**C(H)–C(H_2)–O–H···O(H)–C(C)**	**2.1225**	**3.0825**	**171.137**
	19	C(A)–O–H···O=C(B)	1.7162	2.5920	146.073
		C(C)–O–H···O=C(B)	1.7197	2.5954	145.999
		C(F)–O–H···O=C(G)	1.6989	2.5792	146.514
		C(H)–O–H···O=C(G)	1.6929	2.5754	146.800
		**C(H)–C(H_2)–O–H···O=C(OH)–C(C)**	**1.9875**	**2.9494**	**170.848**
	20	C(A)–O–H···O=C(B)	1.7144	2.5896	145.999
		C(C)–O–H···O=C(B)	1.7102	2.5861	145.949
		C(F)–O–H···O=C(G)	1.7001	2.5786	146.296
		C(H)–O–H···O=C(G)	1.6928	2.5747	146.636
	21	C(A)–O–H···O=C(B)	1.7231	2.5967	145.799
		C(C)–O–H···O=C(B)	1.7153	2.5915	145.992

		C(F)–O–H···O=C(G)	1.7100	2.5896	146.481
		C(H)–O–H···O=C(G)	1.7137	2.5933	146.478

Table S6. Hydrogen Bonds in Sennidin Conformers. PCM optimization in Methanol, σ=0.0001 [Å], σ=0.001 [deg.].

Compound	Conformer	Hydrogen bond	H···O [Å]	O···O [Å]	OHO [deg.]
sennidin A	1	C(A)–O–H···O=C(B)	1.7040	2.5807	146.082
		C(C)–O–H···O=C(B)	1.7029	2.5809	146.215
		C(F)–O–H···O=C(G)	1.7040	2.5807	146.084
		C(H)–O–H···O=C(G)	1.7030	2.5809	146.216
	2	C(A)–O–H···O=C(B)	1.7209	2.5948	145.827
		C(C)–O–H···O=C(B)	1.7148	2.5919	146.075
		C(F)–O–H···O=C(G)	1.7186	2.5927	145.825
		C(H)–O–H···O=C(G)	1.7156	2.5920	146.012
		**C(C)–C(O)–OH···O(H)–C(O)–C(H)**	**1.9499**	**2.8934**	**162.043**
	3	C(A)–O–H···O=C(B)	1.6903	2.5734	146.584
		C(C)–O–H···O=C(B)	1.7131	2.5881	145.834
		C(F)–O–H···O=C(G)	1.6902	2.5734	146.584
		C(H)–O–H···O=C(G)	1.7131	2.5881	145.835
		**C(C)–C(O)–OH···O(H)–C(F)**	**2.1420**	**3.0103**	**147.665**
		**C(H)–C(O)–OH···O(H)–C(A)**	**2.1420**	**3.0103**	**147.666**
	4	C(A)–O–H···O=C(B)	1.7635	2.6208	144.125
		C(C)–O–H···O=C(B)	1.7191	2.5906	145.466
		C(F)–O–H···O=C(G)	1.7634	2.6208	144.126
		C(H)–O–H···O=C(G)	1.7190	2.5906	145.468
		**C(C)–C(O)–OH···O=C(G)**	**1.8464**	**2.8219**	**173.336**
		**C(H)–C(O)–OH···O=C(B)**	**1.8462**	**2.8217**	**173.349**
	5	C(A)–O–H···O=C(B)	1.7217	2.5945	145.680
		C(C)–O–H···O=C(B)	1.7142	2.5909	146.002
		C(F)–O–H···O=C(G)	1.7201	2.5944	145.858
		C(H)–O–H···O=C(G)	1.7156	2.5928	146.093
		**C(H)–C(O)–OH···O=C(OH)C(C)**	**1.7692**	**2.7471**	**170.676**
	6	C(A)–O–H···O=C(B)	1.7270	2.5985	145.534
		C(C)–O–H···O=C(B)	1.7172	2.5922	145.796
		C(F)–O–H···O=C(G)	1.7270	2.5984	145.534
		C(H)–O–H···O=C(G)	1.7172	2.5922	145.796
sennidin B	7	C(A)–O–H···O=C(B)	1.7071	2.5839	146.178
		C(C)–O–H···O=C(B)	1.6999	2.5777	146.135
		C(F)–O–H···O=C(G)	1.7058	2.5820	146.038
		C(H)–O–H···O=C(G)	1.7020	2.5800	146.228
	8	C(A)–O–H···O=C(B)	1.7063	2.5833	146.189
		C(C)–O–H···O=C(B)	1.6993	2.5771	146.148
		C(F)–O–H···O=C(G)	1.7045	2.5810	146.053
		C(H)–O–H···O=C(G)	1.7013	2.5795	146.230
	9	C(A)–O–H···O=C(B)	1.7055	2.5826	146.196
		C(C)–O–H···O=C(B)	1.6878	2.5695	146.461
		C(F)–O–H···O=C(G)	1.7196	2.5923	145.721
		C(H)–O–H···O=C(G)	1.7424	2.6082	145.040
	10	C(A)–O–H···O=C(B)	1.7133	2.5882	145.959
		C(C)–O–H···O=C(B)	1.7047	2.5812	145.958
		C(F)–O–H···O=C(G)	1.7107	2.5845	145.745
		C(H)–O–H···O=C(G)	1.7006	2.5781	146.080

	11	C(A)–O–H···O=C(B)	1.7049	2.5816	146.100
		C(C)–O–H···O=C(B)	1.7015	2.5802	146.296
		C(F)–O–H···O=C(G)	1.6914	2.5738	146.730
		C(H)–O–H···O=C(G)	1.6985	2.5807	146.789
	12	C(A)–O–H···O=C(B)	1.7200	2.5944	145.876
		C(C)–O–H···O=C(B)	1.7133	2.5899	146.052
		C(F)–O–H···O=C(G)	1.7150	2.5930	146.314
		C(H)–O–H···O=C(G)	1.7157	2.5937	146.269
		**C(H)–C(H_2)–O–H···O(H)–C(O)–C(C)**	**2.1580**	**3.1217**	**174.843**
	13	C(A)–O–H···O=C(B)	1.6946	2.5783	146.743
		C(C)–O–H···O=C(B)	1.7170	2.5923	145.916
		C(F)–O–H···O=C(G)	1.6657	2.5574	147.477
		C(H)–O–H···O=C(G)	1.7113	2.5875	146.137
		**C(C)–C(O)–O–H···O(H)–C(F)**	**2.0605**	**2.9542**	**151.445**
		**C(H)–C(H_2)–O–H···O(H)–C(A)**	**2.1192**	**3.0814**	**171.881**
sennidin C	14	C(A)–O–H···O=C(B)	1.7230	2.5934	145.549
		C(C)–O–H···O=C(B)	1.6995	2.5761	146.026
		C(F)–O–H···O=C(G)	1.7625	2.6195	144.083
		C(H)–O–H···O=C(G)	1.7212	2.5947	145.860
		**C(C)–C(O)–O–H···O=C(G)**	**1.8388**	**2.8146**	**172.717**
		**C(H)–C(H_2)–O–H···O=C(B)**	**2.0325**	**2.9933**	**171.078**
	15	C(A)–O–H···O=C(B)	1.7174	2.5932	146.051
		C(C)–O–H···O=C(B)	1.7130	2.5908	146.213
		C(F)–O–H···O=C(G)	1.7166	2.5936	146.189
		C(H)–O–H···O=C(G)	1.7169	2.5940	146.127
		**C(C)–C(O)–O–H···O=C(H)–C(H_2)–C(H)**	**1.6976**	**2.6837**	**170.868**
	16	C(A)–O–H···O=C(B)	1.7247	2.5971	145.649
		C(C)–O–H···O=C(B)	1.7151	2.5911	145.927
		C(F)–O–H···O=C(G)	1.7175	2.5938	146.112
		C(H)–O–H···O=C(G)	1.7167	2.5952	146.288
	17	C(A)–O–H···O=C(B)	1.7071	2.5838	146.116
		C(C)–O–H···O=C(B)	1.7363	2.6025	145.061
		C(F)–O–H···O=C(G)	1.6933	2.5750	146.704
		C(H)–O–H···O=C(G)	1.6853	2.5682	146.690
		**C(H)–C(H_2)–O–H···O=C(B)**	**2.2236**	**3.0967**	**149.681**
	18	C(A)–O–H···O=C(B)	1.7147	2.5874	145.662
		C(C)–O–H···O=C(B)	1.6745	2.5624	147.075
		C(F)–O–H···O=C(G)	1.7025	2.5824	146.544
		C(H)–O–H···O=C(G)	1.7086	2.5873	146.421
		**C(H)–C(H_2)–O–H···O(H)–C(C)**	**2.1214**	**3.0818**	**171.312**
sennidin D	19	C(A)–O–H···O=C(B)	1.7166	2.5922	146.047
		C(C)–O–H···O=C(B)	1.7204	2.5957	145.956
		C(F)–O–H···O=C(G)	1.6994	2.5793	146.475
		C(H)–O–H···O=C(G)	1.6932	2.5755	146.769
		**C(H)–C(H_2)–O–H···O=C(OH)–C(C)**	**1.9889**	**2.9506**	**170.801**
	20	C(A)–O–H···O=C(B)	1.7142	2.5893	145.986
		C(C)–O–H···O=C(B)	1.7108	2.5864	145.907
		C(F)–O–H···O=C(G)	1.7008	2.5789	146.243
		C(H)–O–H···O=C(G)	1.6931	2.5748	146.600
	21	C(A)–O–H···O=C(B)	1.7234	2.5968	145.772
		C(C)–O–H···O=C(B)	1.7159	2.5918	145.963
		C(F)–O–H···O=C(G)	1.7106	2.5900	146.452
		C(H)–O–H···O=C(G)	1.7142	2.5935	146.453

Table S7. The C−C Bond Length, BDE, AIE and SE Calculated for Sennidin Structures.

Sennidine structure	$R_{C\text{-}C}$ [Å]	BDE [kcal·mol⁻¹]	AIE [kcal·mol⁻¹]	SE [kcal·mol⁻¹]
1	1.628	−74.7	−26.9	8.6
2	1.638	−78.3	−31.5	11.0
3	1.662	−84.7	−34.4	5.0
4	1.641	−84.0	−27.6	3.9
5	1.637	−82.7	−32.1	6.4
6	1.631	−76.9	−32.8	9.6
7	1.630	−75.6	−27.7	7.6
8	1.630	−75.7	−27.4	7.4
9	1.635	−78.4	−28.3	7.1
10	1.629	−76.9	−27.6	6.7
11	1.628	−74.9	−27.4	8.1
12	1.630	−77.3	−32.1	10.3
13	1.657	−84.2	−34.7	3.9
14	1.641	−83.2	−29.5	4.0
15	1.629	−83.4	−31.7	5.7
16	1.632	−78.4	−32.8	7.8
17	1.630	−77.9	−25.6	5.9
18	1.639	−78.7	−30.6	5.7
19	1.644	−80.2	−28.4	5.8
20	1.632	−78.1	−28.2	6.1
21	1.630	−75.4	−32.3	10.8

Table S8. Orbital Transition Parameters for Sennidin Derivatives.

Conformer	Energy [cm−1]	Oscylator strength	Contribution of single orbital transition [%]	Orbital transition
1	23829.65	0.0806	98.40	70a→70b
	26002.45	0.0053	98.1	69b→70b
	27239.62	0.0001	82.7	69a→70b
			14.5	69b→71b
			1.2	68b→71a
	28522.66	0.0106	92.7	70a→71a
			3.4	69a→71b
			2.4	69b→70b
	29547.92	0.0052	83.0	69b→71a
			14.7	69a→70b
	23454.59	0.0512	92.2	68b→70b
			3.3	70a→71a
			1.9	69a→71a
	25885.04	0.0346	92.4	69a→71a
			3.1	68b→70b
			2.3	70a→71a
	26304.24	0.2111	72.0	67b→70b
			16.5	66b→70b
			5.2	66a→71a
			2.2	68a→71a

			1.3	67a→71a
	29734.33	0.0062	90.9	68b→71a
			2.5	68a→70b
			2.0	66a→70b
			1.2	66b→71a
	30162.44	0.0174	33.1	66a→70b
			18.2	66b→71a
			17.1	67b→71a
7	23187.45	0.0441	86.1	139a→140a
			11.0	138a→140a
			1.1	137a→140a
	23788.92	0.0747	87.2	138a→140a
			11.1	139a→140a
	25348.96	0.0553	92.7	137a→140a
			2.1	139a→141a
			1.8	138a→141a
	26243.04	0.0616	85.5	139a→141a
			6.3	138a→141a
			2.7	136a→140a
			2.3	137a→141a
			1.3	137a→140a
	26510.84	0.0401	56.4	136a→140a
			29.9	138a→141a
			8.6	139a→141a
			2.5	137a→141a
			1.3	137a→140a
	27028.05	0.0736	58.8	138a→141a
			36.2	136a→140a
			1.3	137a→140a
	28219.08	0.0144	91.5	137a→141a
			2.9	136a→140a
			1.8	136a→141a
			1.0	139a→141a
	29499.62	0.0004	88.1	136a→141a
			3.5	134a→140a
			1.6	137a→141a
			1.6	135a→140a
	29689.09	0.0020	60.0	135a→140a
			21.6	132a→140a
			7.0	133a→141a
			2.0	134a→141a
			1.9	131a→140a
			1.6	131a→141a
			1.2	136a→141a
			1.0	128a→140a
	30071.40	0.0180	43.9	133a→140a
			19.3	134a→140a
			13.0	132a→141a
			7.8	131a→140a
			5.0	136a→141a
			2.8	135a→141a
			1.6	129a→140a
			1.6	131a→141a
11	23490.87	0.0410	58.9	135a→137a

			39.3	136a→137a
	23543.87	0.0626	59.1	136a→137a
			39.7	135a→137a
	25888.46	0.0951	96.7	134a→137a
	27167.32	0.1027	97.0	136a→138a
			8.8	135a→138a
			1.5	133a→137a
			1.1	134a→138a
	27246.58	0.0100	70.5	133a→137a
			19.1	135a→138a
			6.4	136a→138a
	27818.83	0.0868	67.3	135a→138a
			24.8	133a→137a
			4.1	136a→138a
	29402.28	0.0041	88.4	134a→138a
			5.0	132a→137a
			1.6	133a→138a
			1.4	135a→138a
	29654.26	0.0027	70.7	132a→137a
			13.4	131a→137a
			5.4	128a→137a
			4.1	134a→138a
			1.5	127a→137a
	30490.91	0.0153	35.9	128a→137a
			17.2	131a→137a
			13.5	123a→137a
			2.9	127a→137a
			11.2	130a→137a
			1.2	128a→138a
	31546.58	0.0033	55.5	133a→138a
			24.5	131a→137a
			8.2	128a→137a
			4.8	127a→137a
			1.9	134a→138a
17	23491.01	0.0499	97.6	136a→137a
			1.2	135a→137a
	24201.27	0.0399	96.6	135a→137a
			1.2	136a→137a
	25742.95	0.1427	96.5	134a→137a
	26884.92	0.0073	70.3	133a→137a
			24.9	136a→138a
			1.3	135a→137a
	27340.30	0.0134	58.4	136a→138a
			21.3	133a→137a
			16.8	135a→138a
			1.6	134a→138a
	27610.81	0.1721	80.9	135a→138a
			11.3	136a→138a
			5.0	133a→137a
	29170.64	0.0089	85.3	132a→137a
			8.4	134a→138a
			1.3	131a→137a
	29290.08	0.0021	80.6	134a→138a
			9.6	132a→137a

			6.0	133a→138a
			1.7	136a→138a
	31007.43	0.0039	69.7	133a→138a
			7.4	126a→137a
			6.4	131a→137a
			6.0	134a→138a
			2.4	128a→137a
			2.2	127a→137a
			1.2	125a→137a
	31079.69	0.0238	27.0	126a→137a
			20.6	133a→138a
			14.4	131a→137a
			9.5	128a→137a
			7.4	127a→137a
			5.6	130a→137a
			4.5	125a→137a
			3.8	129a→137a
			1.2	132a→137a
			1.2	134a→138a

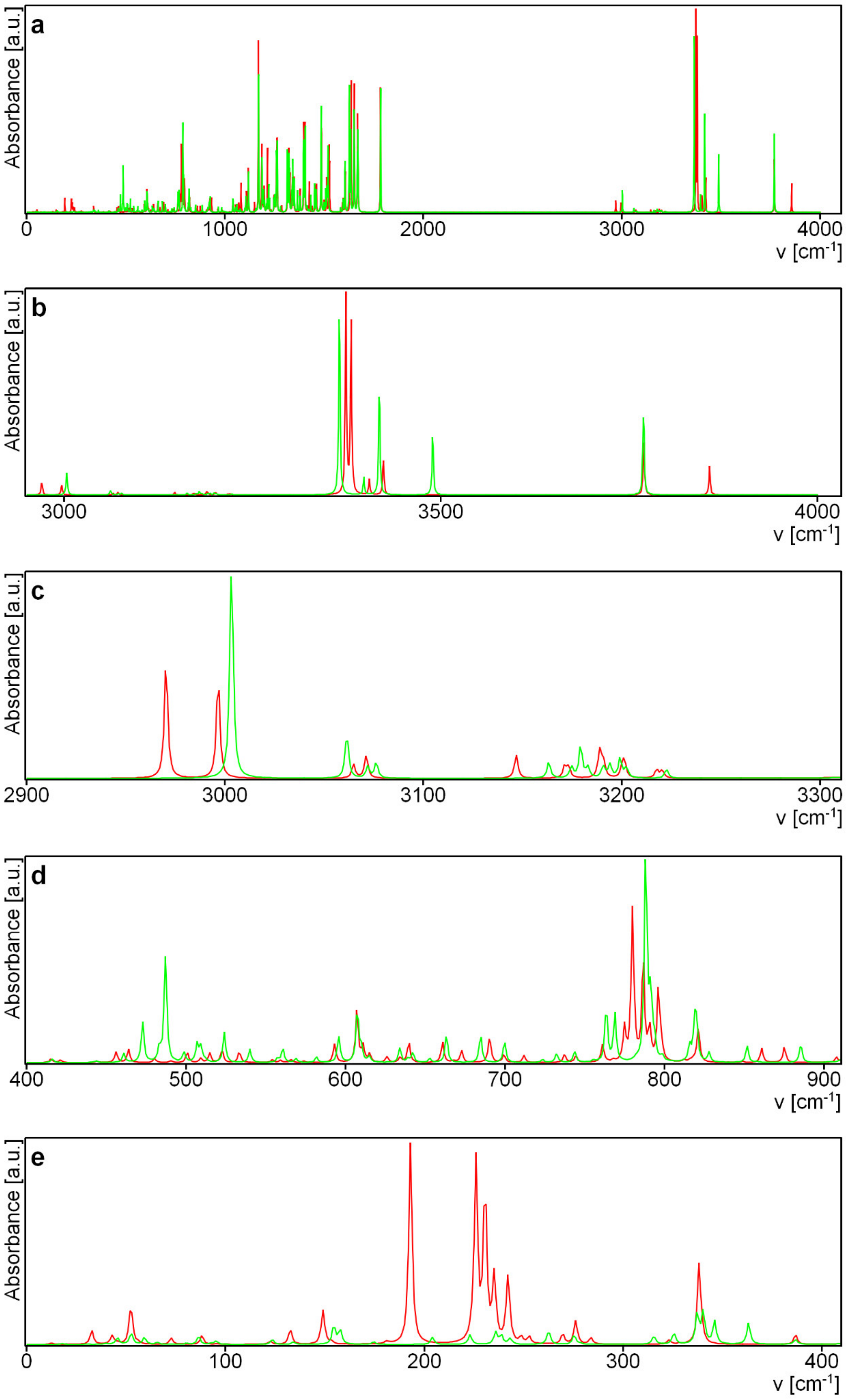

Figure S1. Theoretical IR Spectra for Sennidine C and Sennidine D. Structure **11** – Red, Structure **17** – Green.

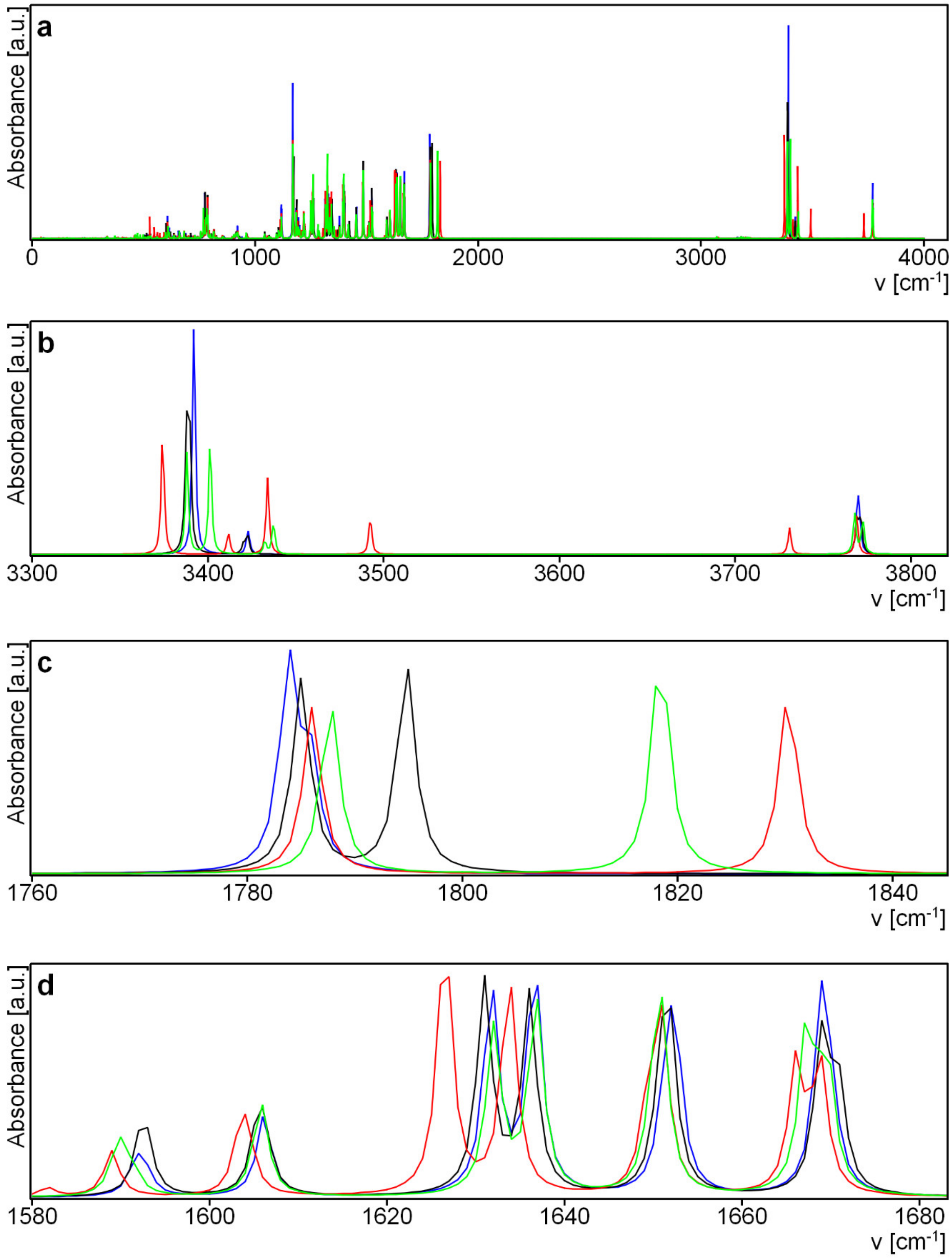

Figure S2. Theoretical IR Spectra for Sennidine B. Structure **7** – Blue, **8** – Black, **9** – Red, **10** – Green.

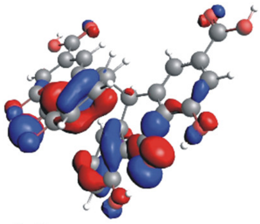

66a

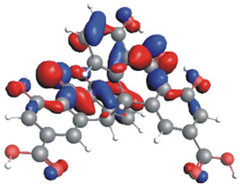

66b

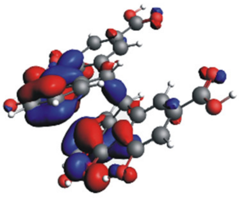

67a

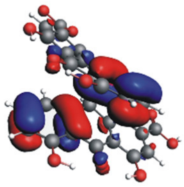

67b

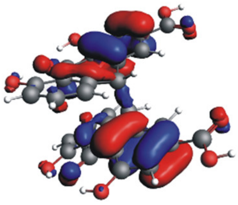

68a

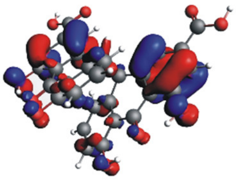

68b

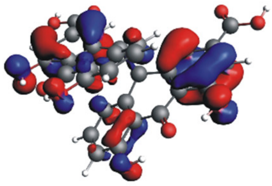

69a

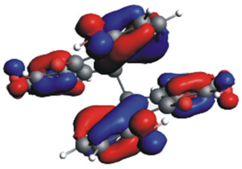

69b

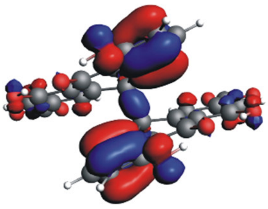

70a

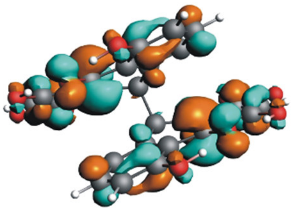

70b

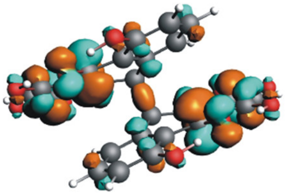

71a

Figure S3. Molecular Orbitals for Sennidine A – Structure **1.**

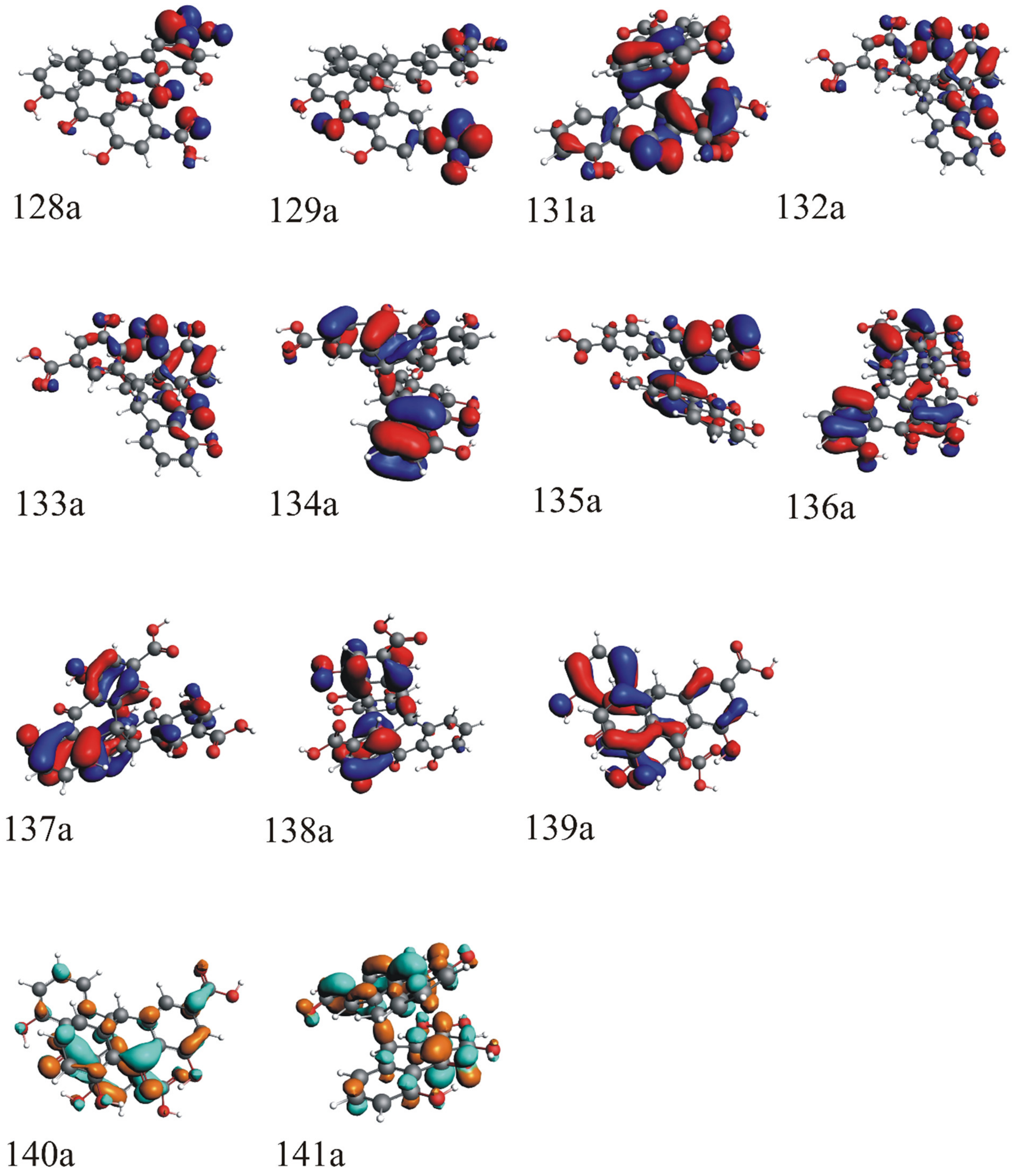

Figure S4. Molecular Orbitals for Sennidine B – Structure **7.**

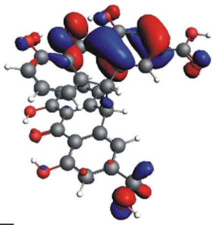

127a

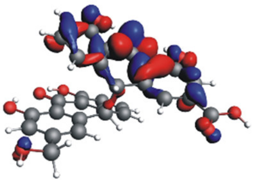

128a

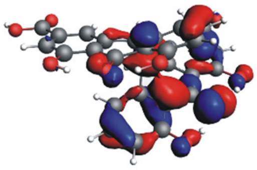

130a

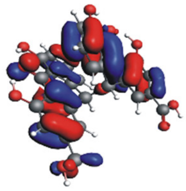

131a

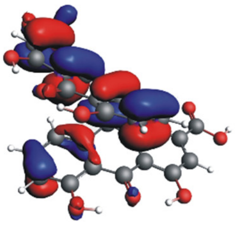

132a

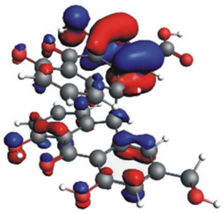

133a

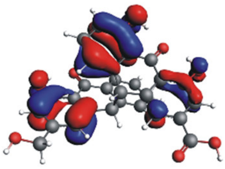

134a

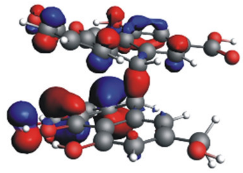

135a

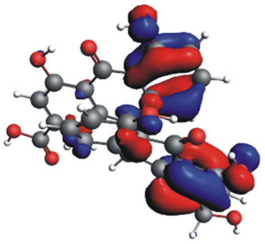

136a

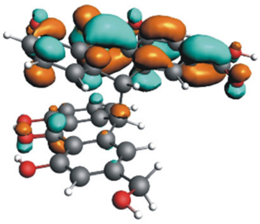

137a

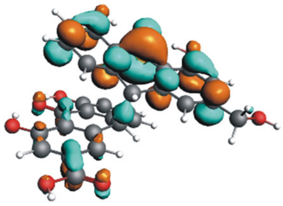

138a

Figure S5. Molecular Orbitals for Sennidine C – Structure **11.**

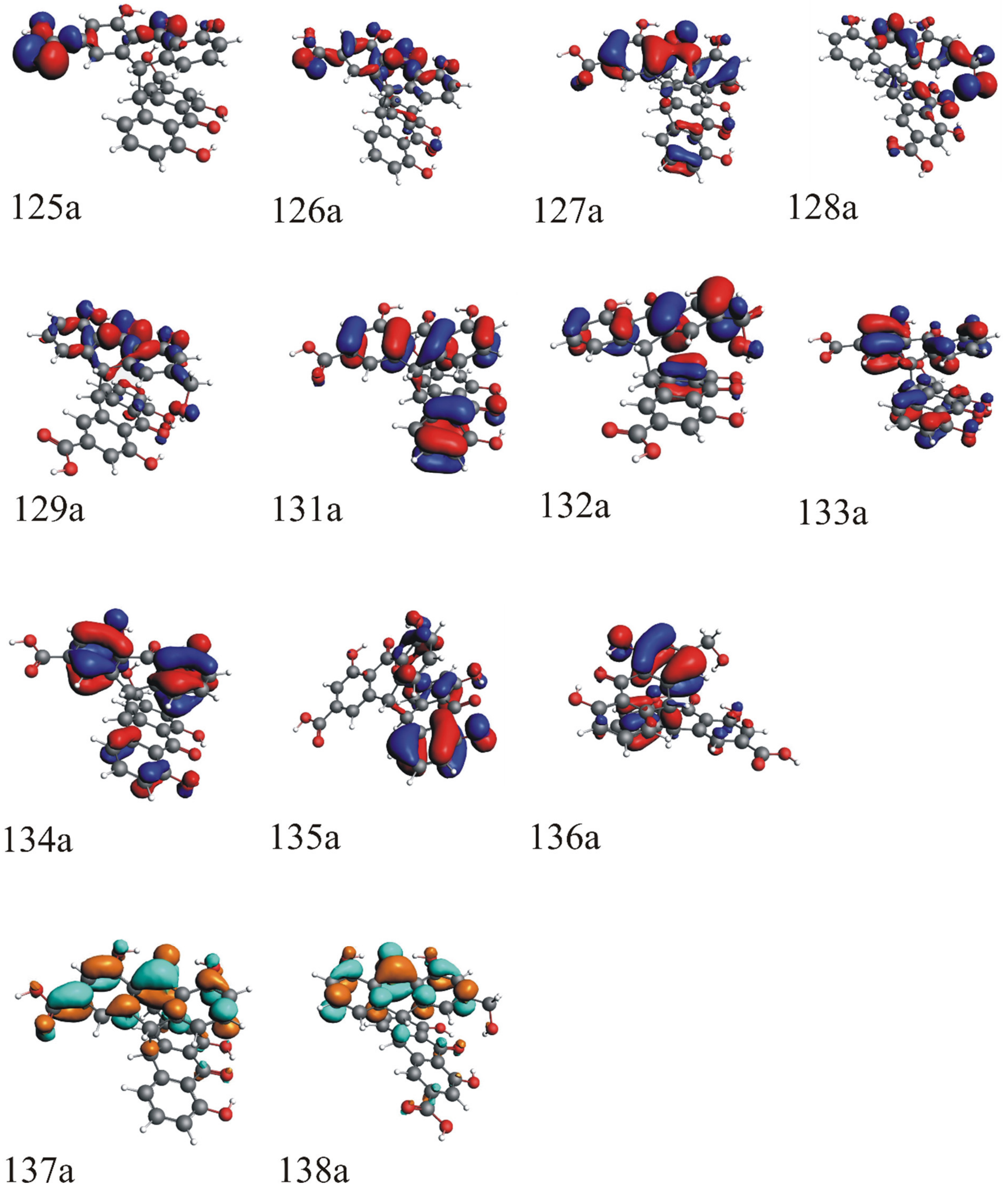

Figure S6. Molecular Orbitals for Sennidine D – Structure **17.**
